# Methods for the economic evaluation of obesity prevention dietary interventions in children: A systematic review and critical appraisal of the evidence

**DOI:** 10.1111/obr.13457

**Published:** 2022-04-27

**Authors:** Sundus Mahdi, Colette Marr, Nicola J. Buckland, Jim Chilcott

**Affiliations:** ^1^ School of Health and Related Research University of Sheffield, Regent Court Sheffield UK; ^2^ Department of Psychology, University of Sheffield Cathedral Court Sheffield UK

**Keywords:** childhood obesity prevention, diet, economic evaluation, systematic review

## Abstract

**Objectives:**

We aim to describe and provide a discussion of methods used to conduct economic evaluations of dietary interventions in children and adolescents, including long‐term modelling, and to make recommendations to assist health economists in the design and reporting of such evaluations.

**Methods:**

A systematic review was conducted in 11 bibliographic databases and the grey literature with searches undertaken between January 2000 and December 2021. A study was included if it (1) was an economic evaluation or modelling study of an obesity‐prevention dietary intervention and (2) targeted 2‐ to 18‐year‐olds.

**Results:**

Twenty‐six studies met the inclusion criteria. Twelve studies conducted an economic evaluation alongside a clinical trial, and 14 studies modelled long‐term health and cost outcomes. Four overarching methodological challenges were identified: modelling long‐term impact of interventions, measuring and valuing health outcomes, cost inclusions and equity considerations.

**Conclusions:**

Variability in methods used to predict, measure and value long‐term benefits in adulthood from short‐term clinical outcomes in childhood was evident across studies. Key recommendations to improve the design and analysis of future economic evaluations include the consideration of weight regain and diminishing intervention effects within future projections; exploration of wider intervention benefits not restricted to quality‐of‐life outcomes; and inclusion of parental or caregiver opportunity costs.

## 1 INTRODUCTION

In 2016, the World Health Organization estimated that over 18% of 5‐ to 19‐year‐olds were affected with overweight or obesity.^1^ The main cause of overweight and obesity is an imbalance between energy consumption and energy expenditure. Diets high in saturated fat and sugar lead to excess energy consumption and contribute to the prevalence and burden of obesity related diseases, including type 2 diabetes mellitus, cardiovascular disease and cancers.^2^, ^3^, ^4^ Obesity‐related health expenditures negatively impact on limited healthcare budgets, costing the UK National Health Service alone over £5.1 billion annually.^5^ Interventions that aim to improve population diet are therefore a priority for policy makers, and evidence on the economics of such interventions is becoming internationally recognized as being crucial to support effective public health policy making.^6^, ^7^, ^8^, ^9^

Health economic evaluations assess additional costs and benefits of an intervention against a comparator (e.g., usual practice). How this is conducted is dependent upon several factors, including the type of economic evaluation approach and whether a healthcare or societal perspective is adopted. Economic evaluations can be conducted alongside clinical trials, where costs and benefits are derived from trial data. Alternatively, clinical effectiveness data can be input into an economic model to derive long‐term cost and benefit outcomes. Where the former provides a cost‐effectiveness estimate using actual trial data, the latter provides long‐term projections of healthcare and societal resource use, costs and associated benefits. The way in which costs and benefits are compared between an intervention and a comparator is dependent on the evaluation framework. There are four main economic evaluation frameworks: (1) cost‐minimization analysis: when different treatment options have equivalent outcomes, therefore the cheapest option is used; (2) cost‐effectiveness analysis: a comparison of additional costs by additional benefits (natural units); (3) cost‐utility analysis: a comparison of additional costs by additional health‐related utilities (e.g., quality‐adjusted life years, disability adjusted life years and health years gained); and (4) cost–benefit analysis: health and/or non‐health benefits are valued in monetary terms (distinctly different to a return on investment which accounts for financial benefits only).^10^

Six systematic reviews have been identified concerning the economics of childhood obesity prevention.^11^, ^12^, ^13^, ^14^, ^15^, ^16^ Most recently, Zanganeh et al. conducted a quality appraisal of the literature^16^ and reviewed the methods adopted within economic evaluations of nutrition and physical activity‐based interventions. However, this study was primarily descriptive in nature and did not provide a critical analysis of the methods, including strengths and limitations, adopted within studies. Oosterhoff et al. also examined key aspects in the design of economic evaluations on school‐based interventions and highlighted key issues and recommendations for future economic evaluations.^14^ However, such reviews have either: lacked a comprehensive search strategy, potentially compromising the inclusion of key texts^11^, ^12^, ^14^; focused on a narrow population group or intervention setting^13^, ^14^; or focused solely on physical activity interventions.^15^

There is currently a lack of consensus on the scope and content of model based economic evaluations for childhood obesity prevention dietary interventions,^17^ leading to variations in assumptions adopted and disparities in final cost‐effectiveness outcomes. This systematic review conducts a comprehensive search and assessment of the literature to develop an understanding of the design of economic evaluations and models, their structure, and methods. The aim of this review is to describe current approaches to the economic evaluation of childhood obesity prevention interventions and make recommendations to assist health economists in the design of such evaluations, with a particular focus on modelling.

## 2 METHODS

### 2.1 Search strategy

The systematic review was registered on PROSPERO (CRD42018115790). It was initially conducted between November 2018 and January 2019 and later updated to December 2021. Bibliographic databases included Medline/PubMed, PsycInfo, Embase, Cochrane Library, Web of Science, SCOPUS, Centre for Reviews and Dissemination (DARE, NHS EED and HTA), EconLit and the Cost Effectiveness Analysis (CEA) Registry. Databases were systematically searched using piloted free text and MeSH terms (Table S1).^18^ In addition, the grey literature was searched using broad terms: “economic evaluation,” “child” and “obesity” and/or “diet.” This included Google, Google Scholar, Grey Literature Report in Public Health and OpenGrey.eu. For Google‐based searches, the first 20 pages of results were examined. Citations of included studies were also searched. Due to the high agreeability rate between the two reviewers in the first set of screening, and resource constraints, only one reviewer screened studies and extracted data from the updated search strategy, unless stated otherwise. Findings from the initial and updated search strategy have been pooled and reported in accordance with PRISMA guidelines.^19^

#### 2.1.1 Inclusion criteria

Criteria for eligible studies included interventions targeting diet and nutrition, either solely or as part of a multi‐component intervention, and with a focus on obesity prevention. Economic studies included economic evaluations alongside trials, or model‐based studies of a single intervention only. The economic analysis of a single intervention, rather than pooled effectiveness data of multiple interventions, was selected due to the high level of heterogeneity found within the design and content of dietary interventions.^20^, ^21^ This also enables an investigation of approaches adopted when single clinical studies are evaluated, allowing easier replication for those taking on a similar approach. No restrictions were placed on the design of the intervention under investigation nor the type of comparator under investigation. The review was restricted to English‐language papers on studies conducted in high‐income countries targeting 2‐ to 18‐year‐olds. This starting age was chosen as children's diets and nutritional needs are comparatively different to subsequent years.^22^, ^23^ No restrictions were placed on clinical or economic study outcomes, which included both direct or proxy measures of obesity prevention. No restrictions were placed on the setting in which interventions were based.

#### 2.1.2 Exclusion criteria

Studies published before the year 2000 were excluded, to ensure the inclusion of up‐to‐date practices, and for pragmatic purposes, given available resources. Modelling studies of hypothetical policies were excluded as they rely on data from multiple intervention studies rather than the evaluation of a single intervention. This review focused on obesity prevention; therefore, weight loss and obesity treatment studies were excluded. Studies targeting niche population and patient groups were also excluded. Finally, studies that measured obesity‐related health conditions with no reference to obesity‐prevention or dietary improvements within their aims were excluded.

### 2.2 Data extraction and quality appraisal

Two data extractions tables were developed, piloted and refined. Two reviewers (S.M. and C.M.) independently extracted data and compared for completeness and accuracy. Any conflicts were discussed until agreement was met.

The Cochrane Public Health Group data extraction and assessment template form^24^ and the CONSORT 2010 checklist^25^ informed the data extraction table of effectiveness studies. Extracted data included study design, intervention description (settings, comparator, strategy, and duration), population, sample size, participant characteristics, attrition rates, missing data management, outcome measures, and results. For extraction of economic evaluation data, the Consolidated Health Economic Evaluation Reporting Standards checklist was adopted.^26^ This included study design, economic outcomes, perspective, time horizon, discount rate, resources and costs, evaluation/modelling methods, databases utilized, methods for dealing with uncertainty, and cost‐effectiveness outcomes.

Following guidance provided by the Centre for Reviews and Dissemination the BMJ 35‐item checklist was used to assess the quality of economic evaluations.^27^ Items designed for the critical appraisal of decision‐analytic models developed for health technology assessment were embedded to cover issues relating to modelling studies.^28^ These included structural assumptions, model type, time horizon, health states and cycle length. Two items from the Paediatric Quality Appraisal Questionnaire were also embedded in order to capture insights into methods for capturing parent and child impacts, including productivity and school absence.^29^ One reviewer assessed the quality of all studies (S.M.) and a second reviewer (C.M.) independently validated 20%.

### 2.3 Data synthesis

A narrative synthesis of the methods used by the economic evaluations was conducted. Characteristics of effectiveness and cost‐effectiveness studies were summarized and details concerning economic evaluation and modelling study methods were identified, compared and set within the context of the broader methods literature. Descriptions of cost‐effectiveness studies, together with reported sensitivity analyses, were used to make recommendations concerning the scope and content of economic evaluations, models and key parameters. Research findings are presented based on a classification of key methodological challenges adapted from Weatherly et al.^30^ Within their paper, several reviews exploring the economics of various public health interventions were investigated in which key methodological challenges were commonly identified across studies: attribution of effects; measuring and valuing outcomes; intersectoral costs and consequences; and equity considerations.

## 3 RESULTS

### 3.1 Literature search: Identification of economic analyses

In the search conducted between December 2018 and January 2019, 13,706 studies were initially identified and 3931 duplicates were removed. One reviewer (S.M.) screened 9775 titles and excluded 7520 studies that were not related to the main inclusion criteria relating to obesity prevention (phase 1 screening). Two reviewers (S.M. and C.M.) independently screened 2255 titles and abstracts (phase 2 screening). There was 71% agreeability between reviewers and after discussions a final number of 45 studies were included for full text screening (phase 3 screening). Seventeen studies were independently included and 22 excluded, whilst the remaining six studies were discussed between reviewers leading to a further two inclusions. One additional paper was identified via the reference list of included studies and included in the review.^31^ In total, 20 papers comprising of 19 separate studies, with one study split across two papers,^32^, ^33^ were included in the systematic review.

In the updated search strategy conducted up to December 2021, 5563 studies were initially identified, and 1336 duplicates were removed. One reviewer (S.M.) screened 4227 titles and excluded 3145 studies that were not related to the main inclusion criteria relating to obesity prevention (phase 1 screening), followed by the screening of 1082 titles and abstracts (phase 2 screening). A final number of 27 studies were included for full text screening (phase 3 screening) whereby a second reviewer (C.M.) screened 30% of full‐texts. There was 100% agreeability between the two reviewers leading to the inclusion of 7 additional studies and the exclusion of 20. No additional papers were identified from references or the grey literature.

**FIGURE 1:**
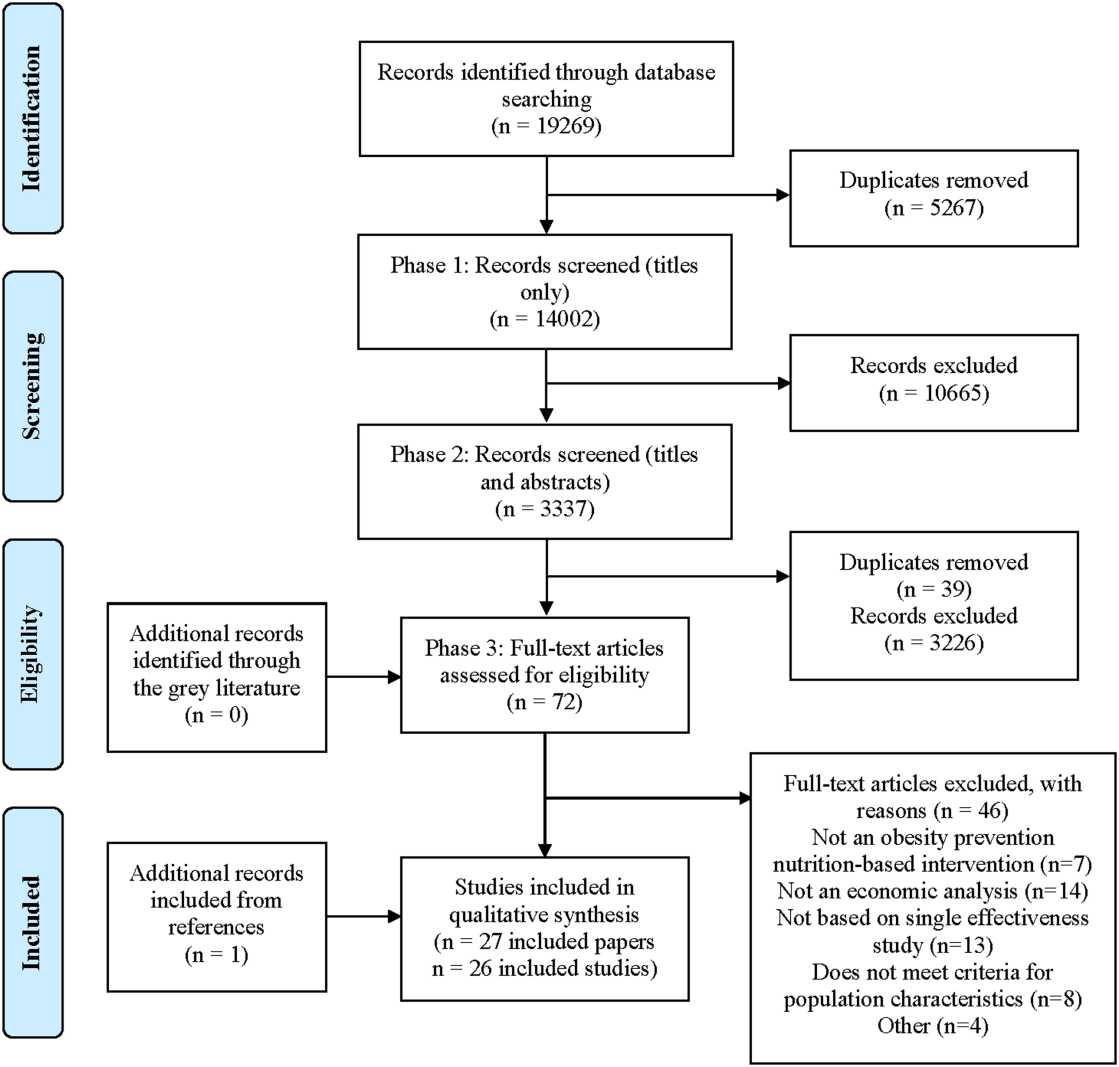
PRISMA flowchart of the study selection process

In total, 27 papers comprising of 26 separate studies were included within this systematic review, and 46 papers were excluded overall after full‐text screening. Main reasons for exclusion included: not an economic analysis (14/46), not based on a single effectiveness study, such as a hypothetical policy (13/46), not meeting criteria for population characteristics, such as age (8/46) and not an obesity prevention nutrition‐based intervention (7/46). Four additional studies were excluded due to there being no intervention comparator, the study was not in the English language, the authors had no access to the paper and study data was previously reported and had been included in the initial search strategy. Figure 1 shows the pooled study selection process.

Quality appraisal outcomes are presented in Table S2 and Table S3. There was 81% concordance in the scoring of studies between the two reviewers. None of the studies fulfilled all the quality criteria and only 19/35 items from the *BMJ* checklist were fulfilled by at least 80% of studies.

### 3.2 Study characteristics

#### 3.2.1 Characteristics of intervention programs

With the exception of two studies, all were school‐based interventions.^32^, ^34^ Four studies self‐identified as school and community‐based interventions,^35^, ^36^, ^37^, ^38^ one targeted day care services^34^ and one was a youth‐camp based intervention.^32^ Eight economic studies were solely based on diet and nutrition interventions,^31^, ^32^, ^34^, ^38^, ^39^, ^40^, ^41^, ^42^ and 15 were nutrition and physical activity based.^31^, ^32^, ^35^, ^37^, ^43^, ^44^, ^45^, ^46^, ^47^, ^48^, ^49^, ^50^, ^51^, ^52^, ^53^ The majority of interventions were compared to a usual practice or “do nothing” scenario.^31^, ^32^, ^34^, ^36^, ^37^, ^39^, ^42^, ^43^, ^45^, ^46^, ^47^, ^49^, ^50^, ^51^, ^53^, ^54^, ^55^, ^56^ One intervention was compared to a control condition where the control school was given money to purchase school equipment,^35^ and four interventions were compared to usual practice with delayed intervention exposure (e.g., waiting list).^38^, ^44^, ^48^, ^57^ One study comprised of three intervention arms,^41^ and another comprised of two.^52^ Intervention arms were compared between each other alongside a usual‐practice comparator, whereas one study compared outcomes between two interventions with no control comparator.^40^ Further intervention characteristics are described in Table S4.

#### 3.2.2 Economic evaluation approach

Table 1 summarizes methods and results of economic analyses. Twelve studies conducted an economic evaluation alongside a clinical trial, of which one conducted a cost‐utility analysis,^43^ eight conducted a cost‐effectiveness analysis^38^, ^41^, ^44^, ^45^, ^48^, ^50^, ^54^, ^57^ and one conducted both.^35^ One study conducted a cost‐consequence analysis^53^ and one conducted both a cost‐effectiveness analysis and cost‐consequence analysis.^34^ Fourteen studies modelled long‐term health and cost outcomes, of which eight applied cost‐utility methods,^31^, ^32^, ^36^, ^46^, ^47^, ^49^, ^52^, ^56^ one conducted a cost–benefit analysis,^39^ and three conducted both.^37^, ^40^, ^55^ One paper conducted a cost‐effectiveness analysis^42^ and one paper conducted a return on investment analysis.^51^ Eight papers adopted Markov decision analytic models,^31^, ^32^, ^36^, ^39^, ^42^, ^46^, ^52^, ^56^ two reported the use of decision trees^55^, ^56^ and the remainder did not refer to the modelling method adopted.^37^, ^40^, ^47^, ^49^, ^51^

#### 3.2.3 Study perspectives and associated intervention costs

Study perspective usually determines cost inclusions. All but one study stated the perspective of the economic analysis.^44^ Fourteen studies claimed a societal perspective,^32^, ^35^, ^36^, ^37^, ^39^, ^40^, ^42^, ^47^, ^50^, ^51^, ^53^, ^54^, ^55^, ^57^ four studies were reported from a healthcare perspective,^31^, ^41^, ^49^, ^56^ and three studies conducted both.^34^, ^40^, ^52^ Three studies also reported from an institutional/school system perspective^45^, ^46^, ^48^ and one from a public sector perspective.^43^

This section will describe how intervention costs were collected and what they consisted of. Discussion of non‐intervention costs are discussed further below. Nineteen studies reported an estimate of staff salaries to implement the intervention, training delivery or training receipt.^34^, ^37^, ^38^, ^40^, ^41^, ^42^, ^44^, ^45^, ^46^, ^47^, ^48^, ^50^, ^51^, ^52^, ^53^, ^54^, ^55^, ^56^, ^57^ Nineteen studies included costs of intervention material and material maintenance (where applicable).^31^, ^34^, ^37^, ^38^, ^39^, ^40^, ^41^, ^42^, ^43^, ^45^, ^48^, ^50^, ^51^, ^52^, ^53^, ^54^, ^55^, ^56^, ^57^ Examples include, water dispensers, books, handouts, sports equipment, food provision, and promotional costs. Ten studies reported additional costs, such as transport, overnight accommodation and utilities.^35^, ^41^, ^42^, ^44^, ^46^, ^50^, ^52^, ^53^, ^54^, ^55^ Two studies reported intervention comparator costs, taking the form of usual school activity costs.^34^, ^50^ Intervention development costs were usually excluded, as this was considered a sunk cost. Five studies excluded school staff costs as the intervention was either embedded within the curriculum, or did not increase staff workload.^33^, ^35^, ^54^, ^55^, ^56^ One study reported the exclusion of unrelated health care costs due to additional years of life,^33^ and out of pocket expenses by individuals due to the intervention.^31^

#### 3.2.4 Discount rates

Discounting of costs and benefits is not required in the case where an intervention lasts 1 year or less, as was the case in eight studies.^34^, ^38^, ^41^, ^48^, ^50^, ^53^, ^54^, ^57^ However, two studies lasting 2 years or over were not discounted.^44^, ^45^ Ten studies indicated a discount rate of 3%,^32^, ^36^, ^37^, ^39^, ^40^, ^42^, ^46^, ^47^, ^51^, ^55^ four studies indicated a discount rate of 3.5%^31^, ^43^, ^49^, ^56^ and one study utilized a discount rate of 5% per annum.^35^ One study applied a 4% discount rate for costs and a 1.5% discount rate for benefits, per annum.^52^ Though typically discount rates are selected based on country‐specific recommendations, seven studies did not justify their discounting choices.^31^, ^35^, ^37^, ^42^, ^43^, ^46^, ^55^

#### 3.2.5 Sensitivity analyses

All but three studies provided details of a sensitivity analysis.^38^, ^44^, ^53^ Probabilistic sensitivity analysis was most often conducted within studies and seeks to explore the impact of parametric uncertainty in the model.^32^, ^33^, ^37^, ^39^, ^40^, ^42^, ^46^, ^47^, ^52^, ^55^ Though the use of probabilistic sensitivity analysis allows description of the parametric uncertainty within economic outcomes, other methods investigate uncertainty of assumptions within the analysis through the variation of one (one‐way sensitivity analysis)^31^, ^32^, ^34^, ^35^, ^36^, ^39^, ^40^, ^41^, ^43^, ^45^, ^47^, ^48^, ^49^, ^50^, ^51^, ^52^, ^54^, ^55^, ^56^, ^57^ or multiple parameters (two‐way or multi‐way sensitivity analysis) at a time.^32^, ^46^, ^47^, ^51^, ^55^ Further details of modelling methods are outlined in Table S5, and the parameters commonly investigated within sensitivity analysis are outlined in Table S6.

**TABLE 1:** Characteristics of economic evaluations

Author, year (country)	Study design; outcomes	Perspective; time horizon; discounting	WTP threshold; key results (base case)
*Economic evaluations alongside trials*			
Adab, 2018 (UK)^43^	CUA; QALYs saved; cases of obesity prevented	Public sector; 18 months; 3.5%/annum	£20–30,000 WTP; £46,083/QALY
Beets, 2018 (USA)^44^	CEA; changes in no. of days F&V, water, deserts and SSBs served	Perspective not declared; 2 years; none declared	No WTP; Cost/child/week for 1 day improvement of F&V = $0.16; SSB = $0.18; Water = $0.28; Dessert improvement = $0.25
Brown, 2021 (Australia)^38^	CEA; intervention cost and ICER per decrease in total and discretionary energy (kJ) packed inside the school lunchbox	Societal; 10 weeks; none	40 AUD WTP = 99% likely cost‐effective; 0.54 AUD per reduction in total lunchbox energy, 0.24 AUD per reduction in kJ from discretionary foods
Conesa, 2018 (Spain)^45^	CEA; cost/no. of obesity cases avoided, decrease in obesity prevalence, BMI unit decrease, BMI z‐score decrease	Institutional; 28 months; none declared	€5/child for 2% reduction in obesity prevalence WTP; €2.4/child/year to reduce the obesity prevalence in boys by 2%
Keszytus, 2013 (Germany)^54^	CEA; change in WC and WtHR	Societal; 1 year; none	€35 WTP; €11.11/1 cm of WC; €18.55/unit of WtHR
Kesztyus, 2017 (Germany)^57^	CEA; cases of obesity averted	Societal; 1 year; none	€123/year parental WTP; Costs/case of incidental abdominal obesity averted varied between €1515–€1993 depending on the size of the observed population, €25.04/child/year
Ladapo, 2016 (USA)^48^	CEA; F&V servings, free/reduced price lunches, full price lunches, all lunches served, snacks served	School; 5 weeks; none	$50,000 WTP; $1.20/additional fruit served during meals, 8.43/additional full priced lunch, $2.11/additional free/reduced‐price lunch, $1.69/reduction in snacks sold
McAuley, 2010 (New Zealand)^35^	CEA and CUA; kg of WGP; HRQoL using the HUI (parental proxy)	Societal; 2 years; 5%/annum	No WTP; no sig diff in HUI scores so did not continue with cost‐utility analysis; $1708/kg of WGP in 7 y/o children; $664/kg of WGP in 13 y/o children
Reeves, 2021 (Australia)^34^	CEA, CCA; service implementation of dietary guidelines	Health sector and modified societal perspective; 1 year; none	No WTP; CEA: intervention dominated, Intervention costs = 4634 AUD, control costs = 7640 AUD, ACER = −2897 AUD
Reilly, 2018 (Australia)^41^	CEA; compliance of healthy canteen policy	Health service delivery; 12 months; none	No WTP; Incremental cost per point increase in proportion of schools reporting adherence: High intensity versus usual: $2982, Medium intensity versus usual: $2627, Low intensity versus usual: $4730. No statistical difference in effectiveness between high and medium intensity
Vieira, 2019 (Portugal)^53^	CCA; comparison of costs and benefits (medical costs averted)	Societal; academic year; none	No WTP; total costs = €7915.53, €36.14/child, €18.18/child (scale‐up), cost of treating obesity = €3849.15/adult with obesity
Wang, 2008 (USA)^50^	CEA; cost/% BF reduction	Societal; 1 year; none	No WTP; $317/0.76% reduction in %BF/student
*Modelling studies*			
An, 2018 (USA)^39^	CBA, MM; cases of childhood overweight prevented, net benefits	Societal; lifetime; 3%/annum	No WTP; $14.5 saved/dollar spent, $174 net benefit/student
Brown, 2007 (USA)^37^	CUA, net monetary benefit; child and projected adult obesity cases averted	Societal; 64 years; 3%/annum	$30,000 WTP; $900/QALY saved, $68,125 base case net‐benefit
Coffield, 2019 (USA)^51^	ROI; comparison of costs accrued over 2 year intervention and costs averted 10 years post intervention	Modified societal; 10 years; 3%/annum	No WTP; intervention cost = $384,717, healthcare spending and productivity losses averted = $581,837, ROI = $1.51/$1 invested
Ekwaru, 2017 (Canada)^46^	CUA, MM; person years of excess body weight, obesity, and chronic disease and QALYs based on 43 health states	School system; 80 years (males), 84 years (female); 3%/annum (costs discounted for 10 years and health outcomes up to 84 years)	$50,000 WTP; $33,421/QALY gained
Graziose, 2017 (USA)^47^	CUA, decision analytic model; reduction in adult obesity, associated medical costs averted and QALYs saved	Societal; 10–40 years; 3%/annum	$50,000 WTP; $275/QALY
Haby, 2006 ‐ benefits^32^ Carter, 2009 – costs^33^ (Australia)	CUA, MM; total age‐specific BMI units (kg/m2); DALYs saved; net cost/DALY saved	Societal; lifetime (100 years); 3%/annum	$50,000 WTP; cost/DALY saved/child: $21,100 (Tamir et al); $5912.50 (Manios et al.); $2800 (James et al.); $38.57 (Gorn et al.)
Mernagh, 2010 (New Zealand)^31^	CUA, MM; cost/QALY	Healthcare; lifetime (100 years); 3.5%/annum	$50,000 WTP; $205,101.45/QALY (APPLE); $168,391.38/QALY (BAEW); $134,252.49/QALY (SNPI)
Kenney, 2019 (USA)^42^	CEA, MM; cost/case of obesity prevented	Modified societal; 10 years; 3%/annum	No WTP; $6542 (95% UI: $1741–$11,918)/case prevented, $0.31(95% UI: $0.15–$0.55) healthcare cost saving/dollar invested
Moodie, 2013 (Australia)^36^	CUA, MM; change in BMI and DALYs averted over the lifetime of the cohort	Societal; lifetime (100 years); 3%/annum	$50,000 WTP; $29,798/DALY saved (intervention population); $20,227/DALY saved (modelling to national level)
Oosterhoff, 2020 (Netherlands)^52^	CUA, MM; cost/QALY	Healthcare and societal; lifetime (100 years); 4%/annum (costs), 1.5%/annum (benefits)	€20,000 WTP; €253.18 healthcare perspective intervention cost/child, €260,152 societal perspective intervention cost, ICER = €19,734
Rush, 2014 (New Zealand)^49^	CUA; BMI and QALYs based on health state preference‐based utilities	Healthcare; lifetime (2–100 years); 3.5%/annum	$50,000 WTP; Project Energize versus 2006 younger children ICER: $30,438; Project Energize versus 2004 older children ICER: $24,690
Te Velde, 2011 (Netherlands)^40^	CUA; DALYs averted/100,000 children, NMB	Healthcare and societal; lifetime; 3%/annum	€19,600/DALY WTP; €5728/DALY averted (prochildren vs. no intervention); €10,674/DALY averted (school guiten vs. no intervention)
Wang, 2003 (USA)^55^	CUA, CBA; cases of adulthood overweight prevented and QALY saved	Societal; 25 years (40–65 years); 3%/annum	$30,000 WTP; $4305/QALY saved
Wyatt, 2016 (UK)^56^	CUA, MM; QALY, life year gained, weight‐related event avoided	NHS and Social Care; 30 years (33–62); 3.5%/annum	£20–30,000 WTP; Dominated

Abbreviations: ACER, average cost‐effectiveness ratio; AUD, Australian dollars; BF, body fat; BMI, Body Mass Index; CAD, Canadian dollars; CBA, cost benefit analysis; CCA, cost‐consequence analysis; CEA, cost‐effectiveness analysis; CI, confidence interval; CUA, cost utility analysis; DALY, disability adjusted life year; F&V, Fruit and vegetables; HRQoL, health related quality of life; HUI, health utility index; IDC, intervention delivery costs; ICER, incremental cost‐effectiveness ratio; MM, Markov Model; NMB, net monetary benefit; QALY, quality adjusted life year; ROI, return on investment; SSB, sugar sweetened beverage; WC, waist circumference; WGP, weight gain prevented; WtHR, waist to height ratio; WTP, willingness to pay; y/o, year old.

### 3.3 Key findings and methodological challenges

Key findings have been categorized into four domains adapted from Weatherly et al.: modelling long‐term impact of interventions; measuring and valuing health outcomes; cost inclusions; and equity considerations.^30^ A critical appraisal of the methods undertaken within cost‐effectiveness studies and key considerations for future economic evaluations of childhood obesity prevention strategies is provided in Table 2. The results are presented as a narrative synthesis and critical appraisal of the methods identified in the economic evaluations.

**TABLE 2:** Critical appraisal of methods undertaken within cost‐effectiveness studies

Methods	Strengths (+) and limitations (−)	Considerations for future evaluations
*Modelling long‐term impact of interventions*		
Inclusion of childhood benefits	(−) Most modelling studies modelled up to the adulthood years. Although children and/or adolescents were targeted within effectiveness studies, the shorter‐term benefits of interventions on child health were not modelled. Inclusion of the shorter‐term benefits may provide useful insights into the immediate benefits, if any, that interventions may have.^52^ ^c^ ^,^ ^d^	The short‐term health and benefit gains from interventions in the childhood and adolescent years should be modelled. Modelling the short‐term outcomes could potentially demonstrate the immediate benefits interventions may have. Such findings may be beneficial to decision makers who will not only see the benefits in the long term but also in the foreseeable future, within their funding cycles.^d^
Two‐step projections	(+) Two‐step probability estimates allow the use of multiple datasets to estimate child to adulthood BMI trajectories. This enables long‐term modelling of outcomes in the absence of longitudinal data.^37^, ^52^, ^55^ Variations of this approach included the transformation of BMI population survey data to approximate future BMI values, in addition to the use of multiple cross‐sectional studies of BMI in children and adults to inform multiple linear regressions based on age effects.^31^, ^32^, ^36^, ^49^ Alternatively, childhood BMI trajectories were used to estimate child weight status up to early adulthood before entering adulthood model.^52^ ^a^ ^,^ ^b^ ^,^ ^d^ (+) Growth trajectories factored covariates such as demographic characteristics and health behaviors, when used to predict future weight status.^42^, ^52^ ^d^ (−) Within studies, the two‐step approach generally assumed a constant relationship between BMI and age and did not account for individual differences.^a^ ^,^ ^d^ (−) There is a danger of using available parameters that are outdated and not reflective of increased obesity rates in the last 20 years.^58^ ^b^	As childhood obesity‐prevention interventions are unlikely to lead to short‐term weight‐related benefits, all modelling studies should aim to carry out long‐term projections of intervention outcomes. In cases where this may not be possible, shorter‐term surrogate markers may be used where they have well‐established links to long‐term outcomes. New data should be incorporated within existing models in cases where evaluations are based on existing model structures. Epidemiological data will need to be constantly updated to provide more accurate estimates that are relevant to the trends faced in present societies.^d^
Multiple logistic regression models for weight status transition probabilities	(+) Inclusion of covariates when obtaining weight status transition probabilities (including age, sex and current weight status) allows for the consideration of expected variability between population subgroups which increases the reliability of predictions.^46^ ^a^ ^,^ ^d^	Weight status transition probabilities should consider the differences in weight status transitions by subgroups.
Adulthood obesity predictions based on childhood intervention outcomes	(+) In cases where there was a lack of evidence to support lifetime projections up to the elderly years, assumptions included maintenance of BMI projections from adulthood, whilst keeping all other environmental factors held constant.^31^, ^49^ Transparency of assumptions adopted are important for purposes of replication and future improvements to model development.^b^ (+) Sensitivity analysis was used to explore intervention effect decay.^31^, ^40^, ^42^, ^46^, ^47^, ^49^, ^51^, ^52^, ^54^, ^57^ This provides valuable insights into the tipping point by which interventions are no longer cost‐effective.^b,d^ However, arbitrary percentages were used due to lack of data.^31^, ^36^, ^46^ (+) Where dietary intake was the primary intervention outcome, evidence on the moderate tracking of fruit and vegetable intake was taken into consideration to form the basis of maintenance of intervention effects, and was varied within sensitivity analysis.^40^ ^a^ ^,^ ^d^ (+) An annual depreciation rate was considered within base case analysis to acknowledge the likelihood that intervention effects diminish with time.^d^ (−) Maintenance of intervention effects was usually not considered within base‐case scenarios of models, despite availability of evidence suggesting the possibility of intervention effects reversing in the long‐term.^59^ There was no evidence from included studies, nor data collected from interventions to evaluate the extent to which weight changes persisted from childhood over time, or whether there were cases of overweight relapse.^37^, ^47^, ^55^ ^b^ ^,^ ^d^	Intervention effects need to be maintained at least up to the point in which disease risks begin to present themselves. Sensitivity analysis can provide insights into the level of maintenance that will need to be achieved for an intervention to be cost‐effective. Whether this is achievable will need to be assessed.^36^ Weight regain after weight‐loss is a prominent obstacle within obesity prevention trials. The possibility of weight regain and diminishing intervention effects needs to be incorporated within models and adjusted within scenario analysis for a more accurate depiction of reality and cost‐effectiveness outcomes.^d^
*Measuring and valuing health outcomes*		
Potential Impact Fractions	(+) BMI was treated as a continuous rather than a categorical variable when considering expected disease due to changes in exposure to the risk factor by BMI unit.^32^, ^36^, ^40^ This is a more accurate reflection of the association between BMI and diseases in comparison to methods that have used weight status to determine disease presence,^31^, ^37^, ^46^, ^47^, ^55^, ^56^ such as is the case with transition probabilities for remaining healthy, developing a weight‐related condition or death.^a^ ^,^ ^d^ (+) Stability was assumed of all incidence and mortality rates from causes other than the diseases included in models.^40^ Although this may not be representative of reality, this ensures that costs and benefits are specifically evaluated for obesity‐related disease states.^a^	The use of BMI as a continuous outcome measure is more accurate than the use of categorical weight status to accurately reflect the associations between weight and disease.^d^
Relative risks of disease incidence and mortality conditional on BMI	(+) Due to low incidence rate data, it was assumed that BMI did not lead to many illness cases before the age of 20 years. Inclusion of illness from age 20 years is considered an improvement in comparison to studies that have investigated disease incidence during older adult years.^31^, ^49^ ^a^ (−) General population incidence rates obtained from a country not related to the study population, was frequently used with no justification.^31^, ^49^ ^b^	All incidence rate data relating to obesity‐related disease should be included within models. The presence of metabolic risk factors, indicative of early‐disease onset, could still lead to increased healthcare resource use and costs. For example, prescription drugs for cholesterol is indicative of an unhealthy diet, despite the absence of overweight or obesity.^60^, ^61^ ^d^ Should obesity‐related parameter estimates be unavailable from the country of intervention under evaluation, the use of another countrys data may be a suitable alternative. Suitability can be determined by factors such as similar lifestyle, diet, obesity prevalence and population characteristics.
QALYs attributed to obesity related diseases	(+) Disutility was not applied to BMI categories in order to avoid potential of double‐counting in cases where someone also had an obesity‐related disease.^31^, ^40^, ^49^ However, the absence of disutility risks underestimating the direct impact of overweight or obesity on health‐related quality of life, in the absence of disease.^62^ ^a^ (−) Obesity‐related disease states were not included in cases where evidence suggests low incidence rates by weight status, thus risking the exclusion of cases of illness within evaluations. ^a^ ^,^ ^d^ (−) Models did not consider different stages of disease severity, but rather the presence or absence of a chronic illness. QALYs attributed to diseases represented the average quality of life over the duration of the illness.^31^, ^49^ It is expected that greater disease severity would be associated with greater BMI^63^, ^64^ and lower HRQoL.^65^, ^66^ ^c^ ^,^ ^d^	Models should incorporate an element of disease severity due to changes in exposure to the risk factor (disease) by BMI unit. This could be embedded within Potential Impact Fractions and taken further to attribute appropriate QALYs by disease severity. ^d^ Given the substantial health benefits and cost‐savings associated with the avoidance of at least one health state, the inclusion of disease states with low incidence rates ought to be incorporated within models.^d^
Disutility for excess weight or chronic disease	(+) Highest disutility value was applied in cases where someone had obesity as well as a chronic illness in order to avoid risk of double‐counting.^46^ This considers both the impacts of HRQoL of obesity and chronic disease.^a^ ^,^ ^d^ (−) Adult based utility decrements had been applied to younger age groups.^46^ HRQoL is typically more impaired within the older than younger years.^67^ Though the consideration of obesity‐related health impacts within the younger years is a progressive step within models, the use of adult‐based data may overestimate the benefits of this.^b^	Where factors may be highly correlated (e.g., obesity and disease states), care should be taken when attributing utilities to weight status in case of double‐counting benefits (or lack thereof). Methods such as applying the highest disutility value between weight status and disease state may be an optimal approach to adopt. Careful consideration needs to be taken when choosing the most appropriate utility values from the literature, including: the population describing the health state (e.g., age, sex), elicitation technique used to derive utility value, sample size and country.^56^
Costs and benefits by weight status	(−) Cost and benefit outcomes were based on long‐term weight status categories (healthy/overweight/obese).^37^, ^47^, ^55^ This assumes that overweight/obesity will impact all individuals equally when outcomes vary by sociodemographics.^42^, ^51^, ^53^, ^68^, ^69^, ^70^ ^a^ ^,^ ^d^	Models should consider covariates within utility and cost estimates. Where there is a lack of existing data, future research should consider the impact of weight status on utility outcomes by sociodemographic classifications.
Consideration of wider intervention effects	(−) Utilities were only captured for direct intervention effects (or for the outcome of interest) and indirect positive effects of the intervention were not considered or measured, potentially leading to an underestimation of cost‐effectiveness. ^a^ (−) Few economic evaluations alongside trials considered child HRQoL using preference‐based outcome measures.^35^, ^43^ There is mixed evidence to suggest that such measures are sensitive enough to detect differences by weight status. ^a^ ^,^ ^d^	Consider evaluating other benefits not directly attributable to the intervention, as not doing so may underestimate the wider intervention benefit. This may not be solely health behaviors, but also individual psychology that may lead to other health benefits as well as cross‐sectoral benefits.^d^ Within the economic evaluation of trials, improved assessment tools need to be designed to detect changes in HRQoL amongst healthy children taking part in a weight gain prevention intervention to protect themselves from future disease.^d^
Choice of outcomes	(−) There was variability in the choice of outcome measures within clinical trials, including objective measures such as BMI,^31^, ^36^, ^37^, ^39^, ^42^, ^46^, ^49^, ^51^, ^52^, ^53^ and subjective measures such as dietary intake.^32^, ^38^, ^40^ Given short‐term follow up of interventions, it is unlikely that any significant changes in BMI or cases of overweight/obesity avoided would have been detected to allow meaningful modelling of long‐term intervention impacts. ^a^ (−) Although there is value in using BMI when assessing health risks of overweight and obesity, this is not the most reliable measure.^71^	In the face of high uncertainty within modelling outcomes, more reliable and objective methods should be adopted to measure dietary or energy intake, for example, doubly labelled water, or the use of adjustment equations for self‐reported data. Where there is a lack of data or evidence from RCTs to support long‐term projections of intervention effects, alternative data sources ought to be considered. Amongst other considerations include non‐experimental data, prospective studies and the application of econometric methodology.^30^ Alternative outcome measures may be better predictors of disease, other than BMI, including waist circumference, or potentially objective dietary intake.^72^
*Cost inclusions*		
Costs converted into rates	(+) Converting costs into rates allows gradual costs of obesity to be factored along with the possibility that not everyone will live the same number of years, hence incurring different amounts of obesity‐related costs.^33^, ^36^ This compares to the use of a block cost estimate for the presence or absence of obesity or related diseases.^37^, ^39^, ^40^, ^47^, ^49^, ^55^, ^56^ The use of rates could help ensure that obesity‐related costs are not overestimated. ^a^ ^,^ ^d^	Conversion of costs into rates may prevent overestimation of obesity‐related costs. The inclusion of covariates, such as age, within equations may further improve estimation of rates though this could introduce further complexity into evaluations.
Costs attributed for overweight and obesity related health states	(−) Not all costs related to all obesity associated health states were included, for example, medical care costs associated with obesity during adolescence and young adulthood. Exclusion of healthcare costs could lead to an underestimation of cost‐effectiveness outcomes. ^b^ ^,^ ^d^ (−) Costs were calculated by weight status/BMI category as opposed to BMI unit, which may overlook cost inclusions.^31^, ^37^, ^39^, ^40^, ^47^, ^49^, ^55^, ^56^ ^a^ (−) Models do not consider the potential changes in healthcare costs at different ages and assume one cost for overweight or obesity. Use of healthcare resources may differ with age, due to greater likelihood of comorbidities, differences in treatment options and plans.^73^ ^a^	Economic analyses ought to expand their inclusion of healthcare costs given the growing evidence of the costs associated with obesity within the childhood years. For example, increased use of GP services and outpatient visits.^74^ These are often overlooked within cost‐effectiveness analyses when considering cost inclusions as cost‐estimates are limited to adulthood healthcare resource use. Consideration of BMI as a continuous variable within evaluations may lead to more accurate estimations of medical and pharmacy costs, expanding to younger age groups.^75^
Wider cost inclusions	(+) Those with obesity may die earlier than healthy weight individuals. The consideration of life expectancy when calculating labor productivity cost estimates could help prevent overestimations of cost‐effectiveness outcomes.^37^, ^55^ In addition, given that weight gain prevention interventions have wider policy implications, they are likely to hold cross‐sectoral costs and consequences. ^a^ (−) Obesity prevention may result in longer years lived, leading to non‐obesity related healthcare costs which was considered by only one study.^52^ ^d^ (−) Opportunity costs of lost time for parents and informal caregivers were rarely considered. Childhood obesity prevention interventions typically involve time commitments from guardians. Cost‐savings from opportunity costs of lost time can also be accrued from the prevention of cases of overweight or obesity (e.g., less visits to the GP with the child). ^a^ ^,^ ^d^ (−) Although some studies had involved parents throughout the roll out of interventions,^32^, ^33^, ^40^, ^43^, ^46^, ^54^, ^57^, ^76^ there was rarely consideration of intervention effects on parents or other family members within models,^51^ potentially leading to an underestimation of the total benefits and cost‐savings of interventions on population health. ^a^ ^,^ ^d^ (−) Studies had not included differential diet costs. Doing so would suggest whether interventions have a negative financial impact on individuals, for example, whether there are financial implications to changes in diets.^d^	Societal or public‐sector perspectives may be more appropriate than a healthcare perspective for obesity prevention interventions, given that public health interventions could lead to numerous cross‐sectoral costs and benefits. Studies taking a societal perspective ought to have broader inclusion of costs relating to societal impacts, including costs of improved diet, parent/caregiver opportunity cost of lost time, work/school absenteeism due to weight‐related sick days for both adult and child.^d^ Spill‐over effects ought to be included within obesity prevention studies, should evidence suggest that interventions have had a positive effect on other family members. ^d^
*Equity considerations*		
Equity considerations	(+) Various subgroup characteristics were explored within economic evaluations, usually conducted through analysis by subgroup and further explored within sensitivity analysis.^31^, ^32^, ^33^, ^36^, ^37^, ^39^, ^40^, ^47^, ^49^, ^52^ 95% confidence intervals were used to guide sensitivity analyses in cases where there was a lack of data sources to guide variations in model parameters.^55^ ^a^ ^,^ ^b^ ^,^ ^d^	Equity ought to be explored within economic evaluations, given the strong link between obesity and socioeconomic status.^58^, ^77^, ^78^ However, studies may not be sufficiently powered to detect meaningful differences between subgroups. Alternative methods such as the use of weights ought to be considered, although these are more computationally complex to administer.^b^

*Note*: All recommendations presented are for where there is data availability.

Abbreviations: BMI, body mass index; HRQoL, health related quality of life; QALY, quality adjusted life year; RCT, randomized controlled trial.

^a^ Discussed within the body of the text.

^b^ Could be improved through further data collection.

^c^ Based on evaluation decision.

^d^ Limitations of cost‐effectiveness studies more generally.

#### 3.3.1 Modelling long‐term impact of interventions

Several challenges in modelling the long‐term impact of interventions were identified. These include the omission of child intervention benefits when adopting lifetime horizons; the approaches used to project long‐term outcomes from childhood to adulthood; and assumptions concerning the maintenance of intervention effects over time. Each of these main issues will now be discussed.

Methodological guidance commonly requires a lifetime horizon in economic analysis. This is particularly relevant in economic evaluations of obesity prevention studies, as many of the benefits of obesity prevention interventions will occur in adulthood. Nevertheless eight studies, all of which conducted economic evaluations alongside trials, based their time horizons on trial duration, which ranged from 5 weeks^48^ to 28 months.^45^ Whereas, modelling studies included cost and benefits over a lifetime,^31^, ^32^, ^36^, ^39^, ^40^, ^49^, ^52^ or truncated analyses at 84,^46^ 65,^37^, ^55^, ^56^ or 40^47^ years. Where truncated lifetime approaches were adopted, authors justified this based on a paucity of long‐term outcomes data. Two studies modelled costs and benefits over a 10‐year time horizon, as this was most relevant for policy makers and due to the long‐term uncertainty regarding intervention effects.^42^, ^51^ One study modelled intervention costs and benefits to cover both the childhood (up to 20 years old) and adulthood years.^52^ However, in most instances health outcomes and associated costs were only modelled throughout adulthood. In doing so, childhood economic benefits of interventions were often overlooked. Emerging research suggests that obesity impacts directly upon child health through early changes in metabolic risk factors^79^, ^80^ and negatively impacts on healthcare resources early on in life.^81^ Failing to include childhood health outcomes risks underestimating the economic benefits of early intervention and increases levels of uncertainty when longer time horizons are considered. Moreover, some decision makers are interested in early outcomes in their own right.^82^ One solution is to present economic outcomes over a selected range of time horizons up to death, allowing the impact on uncertainty to be explicitly communicated.^59^, ^82^ For example, results can be presented for 1, 5, 10, 20, and 50 years.^83^ This will enable the case of investment to be presented, and will demonstrate how interventions can positively impact short‐term outcomes, and avert health complications that may not present until adulthood.

Studies utilized different approaches to modelling long‐term outcomes from childhood‐based effectiveness data. Most commonly, literature was used to obtain childhood to adulthood body mass index (BMI) trajectories.^39^, ^47^, ^56^ In two cases, adult obesity impacts were based directly on rates of child overweight averted in two stages, firstly at 21–29 years, then again at 40 years. This was due to a lack of single progression estimates in published data.^37^, ^55^ Such methods did not account for within‐group differences (e.g., sex) that may result in variability in intervention effects (unlike regression models).^46^ Alternatively, future weight was categorized based on population survey data in annual,^31^, ^49^ or 5‐year increments.^32^, ^36^ When this method was used, the impact of the intervention on mean BMI was subtracted from each simulated individual in the population cohort. This approach often assumed a constant relationship between BMI and age; in addition, subtracting the average decline in BMI across all individuals does not capture the variability of intervention effects across the varying characteristics in the intervention arm (e.g., whether weight gain prevention interventions result in greater BMI reductions amongst individuals with overweight/obesity as opposed to healthy‐weight individuals). Another approach utilized a childhood BMI trajectory to estimate the effect of the intervention on child weight status up to 20 years of age, before entering an adulthood chronic disease model.^52^ In doing so, this method, accommodates assumptions surrounding the immediate and short‐term effects of the intervention. The final approach used regression methods to estimate intervention impact on energy consumption and child weight given age, sex, and height.^32^ This method controls for subgroup differences in weight status transition probabilities and therefore may result in more accurate predictions. Studies that adopted a 10‐year time horizon, either used an annual depreciation rate over 10 years^51^ or shifted children's individual growth trajectories, after exposure to the intervention to estimate future weight status.^42^ Growth trajectory estimates considered demographic characteristics, growth, health behaviors and obesity risk.^42^ In all cases, when deriving parameter estimates, it is imperative that new models adopt the latest epidemiological data in order to accurately reflect the rising trends in overweight/obesity, and associated costs.

Maintenance of intervention effects was assumed within all base‐case analyses, except one.^51^ This is problematic because weight regain after weight loss is a reoccurring problem, meaning that economic outcomes may be overestimated.^84^ One study used an annual depreciation rate of 2.62%, acknowledging the likelihood that intervention effects are not maintained in the long‐term, which reflects clinical findings.^51^ Since data on the maintenance of intervention effects within obesity prevention is currently lacking for children, adult‐based estimates were adopted. To account for intervention effects degrading over time, another study used data on fruit and vegetable consumption from adolescence to young adulthood to justify a 30% lifetime extrapolation of intervention effects within sensitivity analysis.^40^ Other studies examined the impact of declines in intervention effectiveness through sensitivity or scenario analysis,^31^, ^40^, ^46^, ^47^, ^49^, ^52^, ^54^, ^57^ allowing the assessment of parameter and structural uncertainty within the economic evaluations. These analyses led to substantial differences in cost‐effectiveness outcomes in comparison to base‐case scenarios. However, such assumptions were seldomly supported by evidence from longitudinal studies, with approximately half of studies justifying their choice of variables within sensitivity analysis.^32^, ^33^, ^34^, ^35^, ^36^, ^41^, ^42^, ^43^, ^49^, ^50^, ^51^, ^52^, ^54^, ^55^, ^56^, ^57^ Previous work has also demonstrated how incorporating an intervention decay rate can substantially affect the cost‐effectiveness of an obesity intervention,^59^ suggesting the importance of factoring in changes to intervention effectiveness over time.

#### 3.3.2 Measuring and valuing health outcomes

A number of methodological issues associated with measuring and valuing health outcomes were also identified. These related to the methods for associating weight status to disease incidence and mortality, methods for linking disease severity to health utility, the scope of obesity related diseases considered, the wider non‐weight related potential health impacts and the use of current utility instruments.

Inclusion of disease states within models was done through incorporating Potential Impact Fractions, which calculate the proportion change in expected disease or death by change in BMI.^32^, ^36^, ^40^ The use of a continuous risk factor (e.g., BMI) is more accurate than a categorical classification of weight status (e.g., healthy weight/overweight/obese) when predicting disease incidence and mortality rates.^63^, ^64^, ^85^ The use of categorical classifications carries an assumption that all individuals within a classification have the same disease incidence, when there is great variability in BMI within each classification. Other studies applied transition probabilities for remaining healthy, developing a weight‐related condition or death in progressive time intervals.^31^, ^46^, ^52^, ^56^ Although disease states can provide a deeper perspective into the long‐term implications of obesity risks through the incorporation of related costs and consequences, models did not consider how different stages of disease severity could impact upon health utility outcomes. One study considered the impact of increased life years, due to obesity prevention, on age‐related chronic disease.^52^ On the other hand, economic evaluations alongside clinical trials used a variety of clinical outcomes to measure health benefits. This included anthropometric outcomes,^35^, ^50^, ^53^, ^54^ servings of food,^34^, ^44^, ^48^ energy content of packed lunches,^38^ obesity prevalence,^45^, ^57^ and compliance of a healthy canteen policy.^34^, ^41^ Differences in outcomes, without the use of a generic outcome measure such as a quality adjusted life year, increases the difficulty in understanding the significance of the outcome beyond the scope of the immediate study. It also increases difficulty in comparing the cost‐effectiveness of different trials, particularly if there are no standard cut‐off values assigned to the changes in these outcomes, as is the case with quality adjusted life years.

Similarly, when valuing disease states, potential differences in utility by disease severity have not been factored. Some studies used estimates of quality adjusted life years attributed to obesity‐related diseases.^31^, ^49^, ^52^, ^56^ Others used quality adjusted life year measurements associated with obesity in general,^37^, ^47^, ^55^ and one study assigned decrements in health utilities for every year lived with excess weight, obesity or chronic disease within the model. In order to avoid double counting, the highest disutility value was applied in cases where someone had both obesity and a chronic illness.^46^ Future studies ought to consider the inter‐connected relationship between obesity and disease severity, whereby higher BMI classifications are associated with greater health complications, lower health related quality of life and greater healthcare costs.^75^, ^86^ Moreover, perfect health was also assumed for those classified as healthy weight in all studies. This may be an oversight as evidence suggests that health complications occur as a result of unhealthy diets, regardless of weight status.^87^, ^88^, ^89^, ^90^

The number of obesity‐related chronic disease states used within models also varied from four^56^ to fourteen,^31^, ^49^ and commonly included diabetes, cancers, stroke, hypertension, and heart disease. Although disease states were omitted from models,^37^, ^55^, ^56^ potentially due to lack of available data or low incidence rates by weight status, this could exclude relatively rare conditions with a significant economic burden. More simplified models have based cost and benefit outcomes directly on long‐term weight status, whereby cost of illness is associated with overweight/obesity status.^37^, ^42^, ^47^, ^51^, ^53^, ^55^ This assumes that overweight/obesity will impact health states of individuals equally, yet costs may vary by age, sex, socioeconomic status and ethnicity.^68^, ^69^, ^70^, ^91^, ^92^

No study considered the wider non‐weight related potential health gains from improvements in nutrition.^93^ This could underestimate the potential impact of interventions in cases where recipients comply with behavioral changes that have no impact on weight outcomes.^94^ Despite these studies being termed as ineffective,^31^, ^35^, ^43^, ^53^, ^56^ they may have positive effects on comorbidities or non‐health outcomes.^95^, ^96^, ^97^

Two economic evaluations alongside clinical trials used utility instruments (the Health Utility Index^35^ and the Child Health Utility‐9D measure) ^43^ to capture the impacts of obesity prevention interventions. Given children are unlikely to face detrimental health conditions to the same extent as adults, neither intervention led to significant changes in quality adjusted life year outcomes. Indeed, two previous studies have found no statistically significant association between health related quality of life and weight status.^98^, ^99^ Though more recently, a meta‐analysis of international studies found small but significantly lower utility values among 6‐ to 15‐year‐olds with overweight or obesity in comparison to those of healthy weight. This may flag potential differences in the sensitivity of different utility‐based measures amongst different pediatric populations.^100^ Improved assessment tools may need to be designed to detect changes in health related quality of life in weight gain prevention trials among disease‐free children.

#### 3.3.3 Cost inclusions

Limitations involving the inclusion of costs were identified across studies. These comprised of the methods by which costs were included within models, the dismissal of healthcare costs associated with overweight and obesity related health states, and the exclusion of wider costs and potential cost‐savings.

The costs included in an economic evaluation can have a significant impact on the results. Most models incorporated costs associated with either obesity‐based or obesity‐related disease costs.^37^, ^39^, ^40^, ^42^, ^47^, ^49^, ^51^, ^52^, ^55^, ^56^ Mernagh et al. considered health care and medical costs associated with both healthy weight and weight‐related diseases,^31^ whereas others quantified the number of lost sick days for individuals with and without obesity.^37^, ^55^ These methods apply a block total cost for the disease state which may lead to an overestimation of healthcare resources, given that age of death has implications on reduced healthcare use. In the case of Coffield et al., healthcare costs were included if significant associations were found within regressions between healthcare costs and BMI changes.^51^ On the other hand two studies considered gradual healthcare resource use over the lifetime.^33^, ^36^ Carter et al.^33^ and Moodie et al.^36^ converted obesity‐related disease costs for each sex and 5‐year age group into rates for the Australian population. All disease‐specific rates for each sex and age group were summed to give a total obesity‐related disease cost rate. Total cost rates were incorporated into lifetables at each one‐year age group via extrapolation methods. More recently published studies within this review considered medical care costs for both children and adults,^42^, ^51^, ^52^ taking into consideration GP and specialist visits as well as a comparison of medical costs between those with healthy weight and overweight/obesity.^52^ Exclusion of such costs risks the underestimation of cost‐effectiveness outcomes. In addition, only one study incorporated both obesity‐related chronic disease cost and disease costs associated with longer years lived (independent of weight).^52^

Other costs were also not considered by most models. Only three studies incorporated productivity costs by quantifying the number of lost sick days for individuals with and without obesity.^37^, ^52^, ^55^ In addition, 65% of studies did not discuss the relevance of productivity changes to the study question.^31^, ^35^, ^36^, ^37^, ^39^, ^40^, ^43^, ^46^, ^47^, ^50^, ^56^, ^57^ Considering the impact of obesity on productivity,^101^ omitting these costs may lead to a large underestimation of cost‐effectiveness. Moreover, preventing cases of childhood overweight/obesity may lead to a reduction in supervised healthcare visits, and consequently cost‐savings of opportunity costs of lost time. However, only four studies considered opportunity costs of lost time for parents and informal caregivers,^33^, ^36^, ^43^, ^52^ whilst others considered such inclusions within sensitivity analysis,^43^, ^55^ and one study considered school absences^52^ which also holds repercussions to parent/carer workplace productivity costs through increased absenteeism. As such, societal perspectives may be better suited than healthcare perspectives, due to cross‐sector cost implications.

The family unit plays an integral component within childhood obesity‐prevention studies. Childhood obesity prevention interventions are likely to impact the whole household, and not just the recipient child, especially as changes in diet will likely be the result of food purchasing behaviors. This is particularly the case when interventions are not restricted to changes within the school environment, but also involve parents in their administration.^31^, ^32^, ^33^, ^38^, ^40^, ^43^, ^46^, ^51^, ^52^, ^54^, ^57^ As such, childhood obesity prevention trials may lead to spill‐over effects onto other family members,^102^ accruing greater intervention benefits and cost‐savings from disease prevention.^51^ Changes to dietary behaviors can also hold financial repercussions to the household, given that healthier substitutions are more costly than unhealthy, energy‐dense foods.^103^, ^104^, ^105^ However, these were rarely considered within studies.

#### 3.3.4 Equity considerations

The consideration of equity is a key component for economic models of particular relevance for public health interventions.^106^ Health inequalities describe differences in health status between population subgroups associated with economic or social conditions.^107^ Childhood obesity is a worldwide concern that impacts those within disadvantaged groups disproportionately.^58^, ^77^, ^78^ However, less than half of included papers considered equity within their evaluations. Four studies compared outcomes by gender,^32^, ^33^, ^36^, ^39^, ^40^ four studies considered cost‐effectiveness outcomes by ethnicity,^31^, ^37^, ^47^, ^49^ and three considered socioeconomic status,^31^, ^49^, ^52^ of which two identified differences in incremental cost effectiveness ratios between socioeconomic status groups.^49^, ^52^ An intervention that is rejected for scale up as it is not cost‐effective in a general population, may be cost‐effective in a socioeconomically or other disadvantaged group. In such instances, an opportunity to reduce health disparities is missed. Likewise, morbidity and mortality rates may differ by subgroup, potentially leading to inaccurate cost‐effectiveness estimations when parameters are derived from the general population.

## 4 DISCUSSION

This systematic review has assessed the different methods undertaken by studies investigating the cost‐effectiveness of dietary obesity prevention interventions in children and adolescents. It extends previous research by providing a critical synthesis of the strengths and limitations of assumptions adopted within evaluations and provides recommendations for future consideration. Despite the heterogeneity in evaluation approaches, including methods by which adult obesity was predicted from child intervention outcomes, and the choice and methods by which obesity‐related health states, health benefits and related costs were explored, there were key similarities across evaluations. It was generally assumed that intervention effects were maintained, and that the only benefit from interventions was related to obesity prevention. In addition, potential confounding factors were constant from childhood to adulthood and subgroups were rarely included within transition probability calculations, utility estimates and costs. Key considerations for future evaluations are outlined below.

When modelling the long‐term impact of interventions, assuming that intervention effects are maintained from childhood through to adulthood carries a danger of over‐estimating cost‐effectiveness outcomes. Children and adolescents are amenable to changes from the point at which trial data is collected at childhood until adulthood. Therefore long‐term predictions of outcomes may be questionable, especially when intervention effects are known to diminish with time,^108^ and health outcomes relating to the prevention of obesity‐related chronic illness are more likely to present with older age as opposed to childhood. A common approach used within modelling studies was to project adult BMI from child effectiveness outcomes and then calculate the long‐term costs and benefits based on adult parameters. Using sensitivity analysis, the long‐term impact of intervention effectiveness can be varied, though when done, these assumptions are seldomly supported by evidence from longitudinal studies. Recently, Oosterhoff et al. elicited expert opinions on the likely trends in intervention effect maintenance during and after intervention exposure, which were used to model possible BMI trajectories for primary school aged children and adolescents separately. The most popular opinion elicited by experts suggested effect maintenance during intervention exposure, followed by a decay of the relative effects. Results suggested considerable differences between reference intervention effects and expert elicited scenarios.^52^ Brown et al. investigated the impact of effect decay on cost‐effectiveness of obesity prevention interventions in the early years.^59^ Results suggested no health care cost savings if intervention effects decayed to zero after 10 years post‐intervention, in comparison to the substantial cost‐savings should intervention effects be maintained into adulthood. This raises a need for longer follow‐up periods within obesity‐prevention trials to track the maintenance of intervention effects and establish the factors relating to their success or failure over time.^109^, ^110^ Such data could reduce the uncertainty in modelling the long‐term impact of interventions in childhood. Currently, very few studies exist that provide a relative estimate of intervention effect maintenance, though these estimates are within adult populations.^111^ There is also a need to incorporate weight management modules within new or existing cohort or prospective studies, to track the maintenance of intervention effects. Whilst such research may be costly and time‐consuming, it would allow us to better understand the implications of much short‐term intervention research. In developing and validating models of long‐term effects, researchers should explore other reliable sources of data, including commercial providers or existing registries.^112^

We are currently living in an obesogenic environment. Unhealthy diets are more prevalent due to the availability, affordability and accessibility of calorie‐rich foods.^113^, ^114^ Changes need to be made across systems in order to see a significant shift in behavior to reduce obesity prevalence.^2^ Obesity prevention interventions need to be ongoing and sustainable, spanning throughout the life course, tailored to each stage of life where transitions and settings could impact on one's behavior and lifestyle. Whole‐systems approaches may be a potential avenue for exploration, where modifications are made to whole communities.^51^, ^115^ Though this will incur additional substantial costs, the availability of such interventions will ensure that individuals will have constant exposure to obesity prevention strategies, increasing likelihood of long‐term behavior change. However, adopting a life course approach may pose challenges for economic evaluation, as has previously been reported.^116^ For instance, given the number of players involved in implementing a whole of system intervention, spanning across numerous sectors and implemented by both formal (e.g., school) and informal (e.g., parents) parties, tracking of cost inclusion estimates and intervention maintenance costs will be difficult and timely. Until long‐term data is available, there may be uncertainty regarding suitable follow‐up periods for intervention effect size estimates, alongside a suitable comparator. Data collection requirements may be burdensome for community members, and need to be feasible.^117^ There is also a likelihood that intervention benefits will extend beyond child and adolescent recipients,^83^ and may lead to non‐weight related health outcomes. The exposure to multiple behavior change strategies may interact with one another leading to expected or unexpected consequences, which may be difficult to predict and account for.^118^ As such, it has been advised that system dynamic models ought to be utilized in such scenarios to predict changes in system shifts.^116^

Review findings have also highlighted the potential underestimation of cost‐effectiveness outcomes due to the neglect of wider intervention benefits and health outcomes. Engagement in healthier lifestyles may have an impact on child wellbeing,^119^, ^120^ which is seldomly investigated within economic evaluations in children, despite its perceived importance when making decisions on public health investments.^106^ In addition, preference‐based measures may not be sensitive enough to detect changes in health related quality of life amongst children.^121^ New and emerging research is only just starting to investigate child‐based factors that could be incorporated into models. Age‐ and sex‐specific utility values have recently been estimated from the Child Health Utility‐9D measure within an Australian population of 10‐ to 17‐year‐olds. Findings suggested differences in utility values between boys and girls, with significant associations between utilities and BMI *z*‐scores with age.^122^ These findings highlight the importance of factoring in age and sex covariates when modelling long‐term costs and benefits within childhood obesity prevention models. The usability of preference‐based weight‐specific instruments for economic evaluations, such as the Weight‐specific Adolescent Instrument for Economic evaluation (WAItE), have also been investigated. Outcomes have suggested a high correlation between the WAItE, existing generic preference‐based health related quality of life measures, and weight‐specific measures. The WAItE also has an ability to differentiate between weight status and an ability to pick up meaningful changes in health related quality of life.^123^ As such, weight‐specific measures may be better suited for identifying differences in health related quality of life in younger populations.^124^ However, difficulty persists in assessing health related quality of life in healthy individuals who are taking part in weight‐gain prevention interventions to protect long‐term health. This flags the need to develop better measurement tools designed to detect changes in healthy populations.^17^ Difficulty linking health gains to health utilities within children also calls to question the suitability of cost‐utility analysis. Alternative methods such as cost–benefit analysis, where monetary valuations of intervention benefits could be derived via willingness to pay methods,^125^ may have some value.

When considering cost inclusions, various international recommendations suggest the use of a healthcare perspective within base‐case evaluations of health technology assessments.^126^, ^127^ However, rarely do obesity prevention dietary interventions fit within the scope of a healthcare perspective, given they have wider policy implications and cross‐sectoral consequences.^30^ These include school attendance and performance, employment, and productivity, or financial repercussions to individuals due to higher costs of maintaining healthier lifestyles.^17^ The majority of included studies did not factor child‐related productivity costs and their implications, nor healthcare related costs within the childhood years, which may have been due to the lack of data available at the time of evaluation. Recent research investigated the impact of overweight and obesity on school absenteeism in an Australian population of 6‐ to 13‐year‐olds to calculate the indirect repercussions to caregiver lost productivity. Results found that children with obesity missed on average one extra day of school annually in comparison to those without overweight or obesity. This amounted to $338 in indirect carer productivity losses per child.^128^ There has also been an increase in studies investigating childhood obesity related healthcare costs, with findings suggesting substantial medical costs as early as the first 5 years of life,^129^ and greater utilization of general practitioner and specialist weight services.^52^, ^92^, ^130^ Although the inclusion of such costs can be a laborious task, economic evaluations ought to consider cross‐sectoral costs or discuss potential intervention impacts across sectors.

Decision makers have expressed that economic evidence should consider minimizing inequality alongside maximizing efficiency,^106^ and called for a formal weighting of outcomes by population subgroups. Alternative methods may include separate cost‐effectiveness analyses by subgroup, however this has implications for both primary research, for example increased sample sizes to detect subgroup effects, and secondary modelling that would require subgroup specific parameter inputs.^131^

### 4.1 Comparison with previous literature

This paper provides an updated review of the literature. In 2019, Zanganeh et al. published a comprehensive systematic review exploring the methods, study quality and results of economic evaluations for childhood and adolescent obesity interventions.^16^ Similarly, Oosterhoff et al. explored the design, issues and potential solutions to economic evaluations of school‐based lifestyle interventions in 4‐ to 12‐year‐olds.^14^ However, both search strategies were conducted up to early 2017. Fourteen of the included studies within this current paper were published between 2017 and 2021, demonstrating how this area of research is expanding rapidly and the need to regularly update systematic reviews within this domain.

Previous research has acknowledged the shortcomings in methodological recommendations concerning economic evaluations. Frew discusses how current recommendations for economic evaluations are not suited to the evaluation of childhood obesity prevention and outlines key obstacles. These included issues with the conduct of cost utility evaluations, the use of quality adjusted life years for measuring intervention benefits, current issues with cost analyses of interventions and long‐term healthcare savings, and the unsuitability of healthcare perspectives.^83^ More recently, Fattore and colleagues provided recommendations on the type of economic evaluation framework that is most appropriate to conduct concerning nutrition‐based interventions, given intervention design and purpose. They also adopted the use of the Weatherly framework to outline the main challenges in the economic evaluation of nutrition interventions and provided useful recommendations that complement those presented in this paper. For example, when measuring and valuing outcomes, nutrition interventions may generate value far greater than health outcomes and quality adjusted life years alone, including mental and social outcomes. In addition, studies do not consider the potential loss of utility during the intervention period where behavior change is in progress, or the psychological impact changing one's diet may have on an individual.^132^ Despite its strengths, the paper by Fattore and colleagues is not a systematic review of the literature, does not discuss the impact of nutrition interventions on children and adolescents, nor does it focus on obesity prevention. As such, this current systematic review has complemented previous research by not only providing an overview of the characteristics of current economic evaluations, but also delving into a discussion of the evaluation and modelling techniques and assumptions undertaken within this specific area. This has resulted in a comprehensive critical appraisal of the methods and the provision of useful recommendations for future economic evaluations of childhood obesity prevention interventions.

### 4.2 Limitations and recommendations for future research

An early decision was made to exclude studies modelling hypothetical scenarios and those assessing the impact of multiple effectiveness studies. Inclusion of hypothetical studies could have diversified the nature and methods of studies under review. However, closely examining cost‐effectiveness studies of implemented interventions was deemed more suitable for those wishing to undertake a similar research approach. This provides insight into the methods by which economic evaluations and modelling studies are conducted for, and cost‐effectiveness outcomes are compared between, single clinical studies. Similarly, given the growing popularity of childhood obesity prevention interventions within infancy,^13^, ^133^ the exclusion of studies targeting children 2 years old and younger may have led to shortfalls in our understanding of the economics of obesity within the early years and over the life course. In addition, due to a lack of capacity, authors of included studies were not contacted for any unpublished work, which could have minimized publication bias. Most nutrition‐based interventions within this review incorporated a physical activity component. Given that physical activity‐based search terms were not included in the search strategy, as the focus of this review was on nutrition economics, studies whereby diet was a secondary rather than primary focus may have not been identified. Finally, although we adopted recommendations for reporting of systematic reviews by the Centre for Reviews and Dissemination, the data extraction process was extremely timely and resulted in the extraction of more data than was reported. Future systematic reviews may consider the recommendations put forth by Jacobsen and colleagues, whom investigated the key challenges of conducting systematic reviews of economic evaluations, to help focus the reporting of review findings.^134^

Based on the current findings, there are several recommendations for future economic evaluations of childhood obesity prevention interventions. Firstly, interventions ought to consider the possibility of weight regain and diminishing intervention effects within future projections. Where available data is scarce or where there is uncertainty around long‐term intervention effects, comprehensive sensitivity and scenario analysis should be conducted. Secondly, few studies had considered collection of child preference‐based measures, despite the existence of validated measures. A greater focus on the development of outcomes measures sensitive to changes in health related quality of life and wellbeing in healthy children ought to be developed for use within public health prevention interventions, given such interventions focus on promoting healthier lifestyles as opposed to weight loss. Thirdly, very few studies had considered parental or caregiver opportunity costs; non‐obesity related health benefits, including cross‐sectoral costs and consequences should be incorporated. Finally, combating health inequalities is core to public health interventions. It is imperative for studies to explore differences in cost‐effectiveness by subgroups should data permit this.

## 5 CONCLUSIONS

This systematic review provides an overview of economic evaluations of childhood obesity‐prevention dietary interventions. It has extended previous research by providing a deeper understanding of model structures, and the possible assumptions that can be embedded within analyses. In doing so, a number of key methodological challenges were identified within four organizational themes: (1) modelling long‐term impact of interventions; (2) measuring and valuing health outcomes; (3) cost inclusions; and (4) equity considerations. Considerations for future evaluations have been outlined and discussed. The findings of this review should be used to improve methodological decisions and aid the choice of assumptions made within future economic evaluations of childhood dietary interventions.

## CONFLICT OF INTEREST

The authors declare that there is no conflict of interest.

## AUTHOR CONTRIBUTIONS

S.M., N.J.B. and J.C. contributed to the conception of this review; S.M. designed the study; S.M. and C.M. carried out study selection, data extraction and quality appraisal; S.M., C.M. and J.C. carried out analysis and interpretation of data; S.M. drafted the paper; C.M., N.J.B. and J.C. critically revised the paper. All authors have read and approved the content of the review.

## Supporting information


**Table S1** Medline search strategy
**Table S2** Quality Appraisal Summary Data
**Table S3** Quality Appraisal of individual studies
**Table S4** Characteristics of intervention studies
**Table S5** Long‐term modelling methods of cost and benefit outcomes in economic modelling studies
**Table S6** Adjusted parameters within sensitivity analysis
