# Supplementary material for: Methods for the economic evaluation of obesity prevention dietary interventions in children: A systematic review and critical appraisal of the evidence
Source: Obes Rev. 2022 Apr 27;23(9):e13457. doi: 10.1111/obr.13457 (PMC9542346; doi:10.1111/obr.13457)
Supplement: Supplementary file 1 — Table S1 Medline search strategy Table S2 Quality Appraisal Summary Data Table S3 Quality Appraisal of individual studies Table S4 Characteristics of intervention studies Table S5 Long‐term modelling methods of cost and benefit outcomes in economic modelling studies Table S6 Adjusted parameters within sensitivity analysis [file OBR-23-e13457-s001.pdf]

Methods for the economic evaluation of obesity prevention dietary interventions in children: A systematic review and critical appraisal of the evidence

[SUPPORTING INFORMATION]

Sundus Mahdi[1]; Colette Marr[1]; Nicola J Buckland[2]; Jim Chilcott[1]

Author affiliations

[1]School of Health and Related Research, University of Sheffield, Sheffield, UK

[2]Department of Psychology, University of Sheffield, Sheffield, UK

Contact details of corresponding author

Sundus Mahdi – smahdi1@sheffield.ac.uk

School of Health and Related Research, University of Sheffield, Sheffield, UK

Content	Page no.
Search strategy	3-4
Quality appraisal summary data	5-7
Quality appraisal of individual studies	8-12
Characteristics of intervention studies	13-29
Long term modelling methods	30-41
Sensitivity analysis	42-46

TABLE S1 Medline search strategy

#	Searches	Search type	PICOS
**1**	(kindergarten or elementary or pre-school or childhood or children or child* or teen* or adolescen* or kid* or parent or parents or youth or youths or girls or boys or "young person" or "young people").ti,ab.	Search term	Population
**2**	(portion* or purchas* or consum* or sugar* or energy or calorie or calori* or food* or snack* or beverage* or "fast food" or "junk food" or drink or drinks or SSB or soda or sodas or sugar-sweetened or "meal size" or macronutrient* or fruit* or vegetable* or fat or fibre or salt or nutrition* or diet*).ti,ab.	Search term	Intervention
**3**	("body fat" or obese or obesity or adiposity or "body composition" or overweight or weight or BMI or "body mass index").ti,ab.	Search term	Outcome
**4**	("health utility index" or "economic model*" or "economic evaluat*" or cost or "cost benefit*" or cost-benefit* or "cost utilit*" or cost-utilit* or "cost effective*" or cost-effective* or "economic analys*" or economic-analys* or "quality adjusted life year*" or "quality-adjusted life year*" or QALY or "disability adjusted life year*" or "disability-adjusted life year*" or DALY or "life years gained").ti,ab.	Search term	Study design
**5**	adolescent/ or child/	MeSH term	

**6**	Pediatric Obesity/pc [Prevention & Control]	MeSH term
**7**	body weight changes/ or weight gain/ or weight loss/	MeSH term
**8**	Healthy Diet/	MeSH term
**9**	Energy Intake/ph [Physiology]	MeSH term
**10**	models, economic/	MeSH term
**11**	cost-benefit analysis/	MeSH term
**12**	1 or 5	
**13**	3 or 6 or 7	
**14**	2 or 8 or 9	
**15**	13 or 14	
**16**	4 or 10 or 11	
**17**	12 and 15 and 16	
**18**	limit 17 to (english language and humans and "all child (0 to 18 years)" and (classical article or clinical study or clinical trial, all or clinical trial or controlled clinical trial or evaluation studies or government publications or guideline or journal article or meta analysis or observational study or pragmatic clinical trial or randomized controlled trial or "review" or systematic reviews) and last 18 years)	

TABLE S2 Quality Appraisal Summary Data

		Responses: Yes (Y), No (N), Not clear (NC), Not applicable (NA)	***TOTAL N (%)***			
			**Y**	**N**	**NC**	**NA**
		BMJ 35 item checklist				
Study Design	1	The research question is stated	26 (100)	0	0	0
	2	The economic importance of the research question is stated	24 (92)	2 (8)	0	0
	3	The viewpoint(s) of the analysis are clearly stated and justified	24 (92)	2 (8)	0	0
	4	The rationale for choosing the alternative programmes or interventions compared is stated	15 (58)	10 (38)	1 (4)	0
	5	The alternatives being compared are clearly described	23 (88)	3 (12)	0	0
	6	The form of economic evaluation used is stated	25 (96)	0	1 (4)	0
	7	The choice of form of economic evaluation is justified in relation to the questions addressed	24 (92)	2 (8)	0	0
Data collection	8	The source(s) of effectiveness estimates used are stated	26 (100)	0	0	0
	9	Details of the design and results of effectiveness study are given (if based on a single study)	21 (81)	2 (8)	0	3 (11)
	10	Details of the method of synthesis or meta-analysis of estimates are given (if based on an overview of a number of effectiveness studies)	2 (8)	1 (4)	0	23 (88)
	11	The primary outcome measure(s) for the economic evaluation are clearly stated	26 (100)	0	0	0
	12	Methods to value health states and other benefits are stated	16 (61)	0	1 (4)	9 (35)
	13	Details of the subjects from whom valuations were obtained are given	15 (58)	11 (42)	0	0
	14	Productivity changes (if included) are reported separately	3 (11)	2 (8)	0	21 (81)
	15	The relevance of productivity changes to the study question is discussed	5 (19)	17 (65)	0	4 (15)
	16	Quantities of resources are reported separately from their unit costs	13 (50)	13 (50)	0	0
	17	Methods for the estimation of quantities and unit costs are described	22 (85)	4 (15)	0	0

	18	Currency and price data are recorded	26 (100)	0	0	0
	19	Details of currency of price adjustments for inflation or currency conversion are given	9 (35)	17 (65)	0	0
	20	Details of any model used are given	13 (50)	0	0	13 (50)
	21	The choice of model used and the key parameters on which it is based are justified	12 (46)	1 (4)	0	13 (50)
Analysis and interpretation of results	22	Time horizon of costs and benefits is stated	24 (92)	1 (4)	1 (4)	0
	23	The discount rate(s) is stated	19 (73)	4 (15)	0	3 (12)
	24	The choice of rate(s) is justified	9 (35)	7 (27)	0	10 (38)
	25	An explanation is given if costs or benefits are not discounted	3 (12)	2 (8)	0	21 (81)
	26	Details of statistical tests and confidence intervals are given for stochastic data	11 (42)	9 (35)	0	6 (23)
	27	The approach to sensitivity analysis is given	24 (92)	2 (8)	0	0
	28	The choice of variables for sensitivity analysis is justified	15 (58)	9 (35)	0	2 (8)
	29	The ranges over which the variables are varied are stated	18 (69)	7 (27)	0	1 (4)
	30	Relevant alternatives are compared	24 (92)	2 (8)	0	0
	31	Incremental analysis is reported	22 (85)	4 (15)	0	0
	32	Major outcomes are presented in a dissaggregated as well as aggregated form	24 (92)	2 (8)	0	0
	33	The answer to the study question is given	25 (96)	1 (4)	0	0
	34	Conclusions follow from the data reported	24 (92)	2 (8)	0	0
	35	Conclusions are accompanied by the appropriate caveats	24 (92)	2 (8)	0	0
		Phillips et al. (2004) - Modelling studies only				
Structural assumptions	S4	Are the structural assumptions transparent and justified?	13 (50)	0	0	13 (50)
		Are the structural assumptions reasonable given the overall objective, perspective and scope of the model?	12 (46)	1 (4)	0	13 (50)

Model type	S6	Is the chosen model type appropriate given the decision problem and specified causal relationships within the model?	13 (50)	0	0	13 (50)
Time horizon	S7	Is the time horizon of the model sufficient to reflect all important differences between options?	11 (42)	3 (12)	0	12 (46)
		Are the time horizon of the model, the duration of treatment and the duration of treatment effect described and justified?	9 (35)	5 (19)	0	12 (46)
		Has a lifetime horizon been used? If not, has a shorter time horizon been justified?	12 (46)	2 (8)	0	12 (46)
Disease states/ pathways	S8	Do the disease states (state transition model) or the pathways (decision tree model) reflect the underlying biological process of the disease in question and the impact of interventions?	11 (42)	1 (4)	0	14 (54)
Cycle length	S9	Is the cycle length defined and justified in terms of the natural history of disease?	2 (8)	8 (31)	8 (31)	8 (31)
		Pediatric Quality Appraisal Questionnaire				
Cost and Resource use	17	Are opportunity costs of lost time (productivity costs) for parents and informal caregivers measured when required?	5 (19)	18 (69)	0	3 (12)
Outcomes	28	Are school/day-care absences taken into consideration?	1 (4)	25 (96)	0	0

NB: Total percentages may not equal 100% due to rounding.

TABLE S3 Quality Appraisal of individual studies

	Question Number	***Adab et al. 2018***	***An et al. 2018***	***Beets et al. 2018***	***Brown et al. 2007***	***Brown et al. 2021***	***Coffield et al. 2019***	***Conesa et al. 2018***	***Ekwaru et al. 2017***	***Graziose et al. 2017***	***Haby et al. 2006 & Carter 2009***	***Kenney et al. 2019***	***Kesztyus et al. 2013***	***Kesztyus et al. 2017***	***Ladapo et al. 2016***	***McAuley et al. 2010***	***Mernagh et al. 2010***	***Moodie et al. 2010***	***Oosterhoff et al. 2020***	***Reeves et al. 2021***	***Reilly et al. 2018***	***Rush et al. 2014***	***te Velde et al. 2011***	***Vieira et al. 2019***	***Wang et al. 2008***	***Wang et al. 2003***	***Wyatt et al. 2018***
	***BMJ 35 item checklist* Responses: Yes (Y), No (N), Not clear (NC), Not applicable (NA)**																										
Study Design	1	Y	Y	Y	Y	Y	Y	Y	Y	Y	Y	Y	Y	Y	Y	Y	Y	Y	Y	Y	Y	Y	Y	Y	Y	Y	Y
	2	Y	N	Y	Y	Y	Y	Y	Y	Y	Y	Y	Y	Y	Y	Y	Y	Y	Y	Y	Y	N	Y	Y	Y	Y	Y
	3	Y	Y	N	Y	N	Y	Y	Y	Y	Y	Y	Y	Y	Y	Y	Y	Y	Y	Y	Y	Y	Y	Y	Y	Y	Y
	4	Y	Y	Y	Y	Y	N	Y	N	N	Y	Y	N	Y	N	N	NC	Y	Y	Y	Y	N	Y	N	N	N	Y
	5	Y	Y	Y	Y	Y	N	Y	Y	Y	Y	Y	N	Y	Y	Y	N	Y	Y	Y	Y	Y	Y	Y	Y	Y	Y
	6	Y	Y	Y	Y	Y	Y	Y	Y	Y	Y	Y	Y	Y	Y	Y	Y	NC	Y	Y	Y	Y	Y	Y	Y	Y	Y
	7	Y	Y	Y	N	Y	Y	Y	Y	Y	Y	Y	Y	Y	Y	Y	Y	N	Y	Y	Y	Y	Y	Y	Y	Y	Y
Data collection	8	Y	Y	Y	Y	Y	Y	Y	Y	Y	Y	Y	Y	Y	Y	Y	Y	Y	Y	Y	Y	Y	Y	Y	Y	Y	Y

Question Number	***Adab et al. 2018***	***An et al. 2018***	***Beets et al. 2018***	***Brown et al. 2007***	***Brown et al. 2021***	***Coffield et al. 2019***	***Conesa et al. 2018***	***Ekwaru et al. 2017***	***Graziose et al. 2017***	***Haby et al. 2006 & Carter 2009***	***Kenney et al. 2019***	***Kesztyus et al. 2013***	***Kesztyus et al. 2017***	***Ladapo et al. 2016***	***McAuley et al. 2010***	***Mernagh et al. 2010***	***Moodie et al. 2010***	***Oosterhoff et al. 2020***	***Reeves et al. 2021***	***Reilly et al. 2018***	***Rush et al. 2014***	***te Velde et al. 2011***	***Vieira et al. 2019***	***Wang et al. 2008***	***Wang et al. 2003***	***Wyatt et al. 2018***
9	Y	Y	Y	Y	Y	Y	Y	N	Y	NA	N	Y	Y	Y	Y	NA	Y	Y	Y	Y	Y	NA	Y	Y	Y	Y
10	NA	NA	NA	NA	NA	NA	NA	NA	NA	Y	NA	NA	NA	NA	NA	N	NA	NA	NA	NA	NA	Y	NA	NA	NA	NA
11	Y	Y	Y	Y	Y	Y	Y	Y	Y	Y	Y	Y	Y	Y	Y	Y	Y	Y	Y	Y	Y	Y	Y	Y	Y	Y
12	Y	Y	NA	Y	NA	Y	NA	Y	Y	Y	Y	NA	NA	NA	Y	Y	Y	Y	NA	NA	NC	Y	Y	NA	Y	Y
13	Y	N	N	Y	Y	N	N	Y	Y	N	N	Y	Y	Y	Y	N	N	Y	Y	Y	N	Y	Y	N	N	Y
14	NA	Y	NA	N	NA	N	NA	NA	NA	NA	NA	NA	NA	NA	NA	NA	NA	Y	NA	NA	NA	NA	NA	NA	Y	NA
15	N	N	NA	N	N	Y	NA	N	N	Y	N	NA	N	NA	N	N	N	Y	N	N	Y	N	N	N	Y	N
16	N	Y	N	Y	N	N	N	N	Y	N	N	Y	Y	Y	Y	N	N	Y	Y	N	N	N	Y	Y	Y	Y
17	Y	Y	Y	Y	Y	Y	N	N	Y	Y	N	Y	Y	Y	Y	Y	Y	Y	Y	Y	N	Y	Y	Y	Y	Y
18	Y	Y	Y	Y	Y	Y	Y	Y	Y	Y	Y	Y	Y	Y	Y	Y	Y	Y	Y	Y	Y	Y	Y	Y	Y	Y

	Question Number	**Adab et al. 2018**	**An et al. 2018**	**Beets et al. 2018**	**Brown et al. 2007**	**Brown et al. 2021**	**Coffield et al. 2019**	**Conesa et al. 2018**	**Ekwaru et al. 2017**	**Graziose et al. 2017**	**Haby et al. 2006 & Carter 2009**	**Kenney et al. 2019**	**Kesztyus et al. 2013**	**Kesztyus et al. 2017**	**Ladapo et al. 2016**	**McAuley et al. 2010**	**Mernagh et al. 2010**	**Moodie et al. 2010**	**Oosterhoff et al. 2020**	**Reeves et al. 2021**	**Reilly et al. 2018**	**Rush et al. 2014**	**te Velde et al. 2011**	**Vieira et al. 2019**	**Wang et al. 2008**	**Wang et al. 2003**	**Wyatt et al. 2018**
	19	Y	Y	N	N	N	Y	N	Y	N	N	N	N	N	N	N	Y	Y	Y	N	N	N	Y	N	Y	N	N
	20	NA	Y	NA	Y	NA	NA	NA	Y	Y	Y	Y	NA	NA	NA	NA	Y	Y	Y	NA	NA	Y	Y	NA	NA	Y	Y
	21	NA	Y	NA	Y	NA	NA	NA	Y	Y	Y	N	NA	NA	NA	NA	Y	Y	Y	NA	NA	Y	Y	NA	NA	Y	Y
Analysis and interpretation of results	22	Y	Y	NC	Y	Y	Y	N	Y	Y	Y	Y	Y	Y	Y	Y	Y	Y	Y	Y	Y	Y	Y	Y	Y	Y	Y
	23	Y	Y	N	Y	NA	Y	N	Y	Y	Y	Y	Y	Y	N	Y	Y	Y	Y	NA	Y	Y	Y	NA	N	Y	Y
	24	N	Y	NA	N	NA	Y	NA	N	Y	Y	N	NA	NA	NA	N	N	Y	Y	NA	NA	Y	Y	NA	NA	N	Y
	25	NA	Y	N	NA	NA	NA	NA	NA	NA	NA	NA	Y	Y	N	NA	NA	NA	NA	NA	NA	NA	NA	NA	NA	NA	NA
	26	Y	N	N	Y	Y	N	Y	N	Y	Y	Y	NA	NA	NA	NA	N	Y	Y	Y	N	NA	N	N	NA	Y	N
	27	Y	Y	N	Y	Y	Y	Y	Y	Y	Y	Y	Y	Y	Y	Y	Y	Y	Y	Y	Y	Y	Y	N	Y	Y	Y
	28	Y	N	NA	N	N	Y	N	N	N	Y	Y	Y	Y	N	Y	N	Y	Y	Y	Y	Y	N	NA	Y	Y	Y

	Question Number	***Adab et al. 2018***	***An et al. 2018***	***Beets et al. 2018***	***Brown et al. 2007***	***Brown et al. 2021***	***Coffield et al. 2019***	***Conesa et al. 2018***	***Ekwaru et al. 2017***	***Graziose et al. 2017***	***Haby et al. 2006 & Carter 2009***	***Kenney et al. 2019***	***Keszytus et al. 2013***	***Keszytus et al. 2017***	***Ladapo et al. 2016***	***McAuley et al. 2010***	***Mernagh et al. 2010***	***Moodie et al. 2010***	***Oosterhoff et al. 2020***	***Reeves et al. 2021***	***Reilly et al. 2018***	***Rush et al. 2014***	***te Velde et al. 2011***	***Vieira et al. 2019***	***Wang et al. 2008***	***Wang et al. 2003***	***Wyatt et al. 2018***
	29	Y	Y	NA	Y	N	Y	N	Y	Y	N	N	Y	Y	N	Y	Y	Y	Y	Y	N	Y	Y	N	Y	Y	Y
	30	Y	Y	N	Y	Y	N	Y	Y	Y	Y	Y	Y	Y	Y	Y	Y	Y	Y	Y	Y	Y	Y	Y	Y	Y	Y
	31	Y	Y	Y	N	Y	N	Y	Y	Y	N	Y	Y	Y	Y	Y	Y	Y	Y	Y	Y	Y	Y	N	Y	Y	Y
	32	Y	N	Y	Y	Y	Y	N	Y	Y	Y	Y	Y	Y	Y	Y	Y	Y	Y	Y	Y	Y	Y	Y	Y	Y	Y
	33	Y	Y	Y	Y	Y	Y	Y	Y	Y	Y	Y	Y	Y	Y	Y	Y	Y	Y	Y	Y	Y	Y	N	Y	Y	Y
	34	Y	Y	Y	Y	Y	Y	Y	Y	Y	Y	Y	Y	Y	Y	Y	Y	N	Y	Y	Y	Y	Y	N	Y	Y	Y
	35	Y	Y	Y	Y	Y	Y	Y	Y	Y	N	Y	Y	Y	Y	Y	Y	Y	Y	Y	Y	Y	Y	N	Y	Y	Y
		Phillips et al. (2004) - Modelling studies only																									
Structural assumptions	S4	NA	Y	NA	Y	NA	NA	NA	Y	Y	Y	Y	NA	NA	NA	NA	Y	Y	Y	NA	NA	Y	Y	NA	NA	Y	Y
		NA	N	NA	Y	NA	NA	NA	Y	Y	Y	Y	NA	NA	NA	NA	Y	Y	Y	NA	NA	Y	Y	NA	NA	Y	Y
Model type	S6	NA	Y	NA	Y	NA	NA	NA	Y	Y	Y	Y	NA	NA	NA	NA	Y	Y	Y	NA	NA	Y	Y	NA	NA	Y	Y

	Question Number	Adab et al. 2018	An et al. 2018	Beets et al. 2018	Brown et al. 2007	Brown et al. 2021	Coffield et al. 2019	Conesa et al. 2018	Ekwaru et al. 2017	Graziose et al. 2017	Haby et al. 2006 & Carter 2009	Kenney et al. 2019	Kesztyus et al. 2013	Kesztyus et al. 2017	Ladapo et al. 2016	McAuley et al. 2010	Mernagh et al. 2010	Moodie et al. 2010	Oosterhoff et al. 2020	Reeves et al. 2021	Reilly et al. 2018	Rush et al. 2014	te Velde et al. 2011	Vieira et al. 2019	Wang et al. 2008	Wang et al. 2003	Wyatt et al. 2018
Time horizon	S7	NA	Y	NA	Y	NA	Y	NA	Y	N	Y	Y	NA	NA	NA	NA	Y	Y	Y	NA	NA	Y	Y	NA	NA	N	N
		NA	Y	NA	N	NA	Y	NA	Y	N	N	Y	NA	NA	NA	NA	Y	N	Y	NA	NA	Y	Y	NA	NA	N	Y
		NA	Y	NA	Y	NA	Y	NA	Y	N	Y	Y	NA	NA	NA	NA	Y	Y	Y	NA	NA	Y	Y	NA	NA	N	Y
Disease states/ pathways	S8	NA	N	NA	Y	NA	NA	NA	Y	Y	Y	NA	NA	NA	NA	NA	Y	Y	Y	NA	NA	Y	Y	NA	NA	Y	Y
Cycle length	S9	NA	N	NA	N	NA	NA	NA	NC	N	N	NA	NA	NA	NA	NA	Y	NC	N	NA	NA	N	N	NA	NA	N	Y
		Pediatric Quality Appraisal Questionnaire																									
Cost and Resource use	17	Y	NA	N	N	N	Y	N	N	N	Y	N	N	N	N	N	N	Y	Y	N	NA	NA	N	N	N	N	N
Outcomes	28	N	N	N	N	N	N	N	N	N	N	N	N	N	N	N	N	N	Y	N	N	N	N	N	N	N	N

Responses: Yes (Y), No (N), Not clear (NC), Not applicable (NA)

See Table S2 Quality Appraisal Summary Data for full questions/criteria alongside question numbers

TABLE S4 Characteristics of intervention studies

Author (year)	Study design; duration (reference)	Intervention; components	Sample; population; age group	Outcome measures	Key results
**Adab et al. (2018)[1]**	Cluster RCT; 12 months	WAVES trial; healthy eating, physical activity, parental engagement, signposting.	N=2462 (baseline); primary schools in West Midlands, UK; 5-6 year olds	BMI-z (UK 1990 reference curves), WC, skinfold thickness, %BF, dietary intake, quality of life (CHU9D)	Not significant; mean BMI-z difference between control and intervention arms at 18 months = -0.027 (95% CI = -0.137 to 0.083)
**An et al. (2018)[2]**	Quasi experimental study; 4 years [3]	Promote plain water consumption; installation of water dispensers.	N=1,065,562 (baseline); public elementary schools, New York; kindergarten to 8th grade	BMI	Significant BMI-z reduction of 0.025 in boys (95% CI = -0.038 to -0.011).<br>Significant BMI-z reduction of 0.022 in girls (95% CI= -0.035 to -0.008).<br><br>Risk reduction of childhood overweight by 0.9% among boys (95% CI= 0.015 to 0.003) and 0.6% among girls (95% CI= 0.011 to 0.000).
**Beets et al. (2018)[4]**	RCT with one-year delayed treatment group; 2 years [5]	After school programme; serves healthy foods and encourages physical activity	N=2663 (baseline); after school programmes, South Carolina; 5-12 years	Foods and beverages served	Increased number of days/week for servings of fruits/veg: 0.6 vs 1.7 (delayed group), 0.6 vs 4.4 (immediate group), OR=3.8, 95% CI=1.45 to 9.95.

					SSBs: 1.2 vs 0.2 (delayed group), 3.2 vs 0 (immediate group), OR=0.05, 95% CI=0.02 to 0.13. Desserts: 2.9 vs 0.6 immediate group), OR=0.1, 95% CI=0.03 to 0.33.
**Brown et al. (2007)*[6]**	Untreated, matched control group design with repeated dependent pretest and posttest samples; quasi experimental design; 4 years[7]	CATCH programme; nutrition and physical activity embedded within curriculum, family involvement	N=896 (baseline); elementary schools, El Paso, Texas; 8-11 years	Risk of overweight or obesity using BMI percentiles (+85th or +95th), percentage of fat and sodium in school lunches, physical activity.	No effect of CATCH on anthropometry<br>Girls: Rate of increased overweight risk in CATCH schools significantly lower (2%) to controls (13%).<br>Boys: Rate of increased overweight risk significantly lower (1%) in CATCH schools compared to controls (9%).
**Brown et al. (2021)[8]**	2x2 factorial cluster randomised controlled trial; 10 weeks[9]	SWAP IT intervention; encouraged parents to swap lunchbox discretionary food items to healthier alternatives through a school communication	N=778 (baseline intervention), N=991 (baseline control); primary schools, New South Wales, Australia; 5-12 years	Items packed in lunchbox (mean kJ), mean total and percent energy from foods that align with Australian Dietary Guidelines and discretionary food items.	Reduction in total energy from school lunchbox: -131.61 kJ, CI = -317.26, 54.05, p = 0.16; reduction in energy from discretionary foods: -211.61 kJ, CI = -426.16, 2.95, p = 0.05; increase in energy from healthier everyday food: 83.13 kJ, CI = 2.65, 163.61, p = 0.04

		app, educational component			
**Coffield et al. (2019)*[10]**	Non-randomised controlled trial; 2 years[11]	Shape Up Sommerville (SUS); Diet and PA, whole-system approach targeting school (e.g. school food service), home (e.g. parent education) and community (e.g. "approved" restaurants) environments	N=1028; schools, home and community, Massachusetts, USA; grades 1-3.	BMI-z from height and weight.	BMI-z intervention relative to controls: -0.057 (95% CI: -0.08, -0.04)<br><br>Self-reported BMI of intervention parents, relative to control group: -0.411 (95% CI: -0.725, -0.097)
**Conesa et al. (2018)[12]**	Randomised, parallel, controlled primary school-based obesity prevention intervention; 28 months [13]	Educacio en Alimentacio (EdAI) program; educational activities promoting nutrition	N=2350; primary schools, Catalonia, Spain; 7-8 years	Prevalence of obesity (primary), changes in BMI z-score, WC and incidence & remission of excess weight (secondary)	Obesity prevalence decreased by 2.02% in intervention group and increased by 0.44% in control group.<br>Boys: 4.39% difference in obesity prevalence (95% CI: 3.48 – 5.30%, p = .01).<br>Intervention boys had sig. reduction of −0.24 units in the BMI z-score compared with the control group.<br>Girls: no sig. differences
**Ekwaru et al. (2017)[14]**	Observational; 2 years [15]	The Alberta Project Promoting active Living and healthy	N=7850; elementary schools,	BMI, dietary intake (FFQ), physical activity	Sig difference in changes in calorie intake (mean -212kcal, 95% CI: -

		Eating in Schools (APPLE); nutrition and physical activity, parental engagement	Alberta, Canada; grade 5 (~10 years)		315 to -109) of APPLE students compared to control.<br>2.2% reduction in obesity prevalence between 2008-2010 among APPLE schools compared to 2.8% increase in control.<br>APPLE Schools estimated to reduce odds of obesity over normal weight by 0.723 times per year (OR = 0.723, 95%CI: 0.553 to 0.946).
**Graziose et al. (2017)[16]**	Cluster RCT; 1 year	Food, Health & Choices (FHC) program; curriculum embedded lessons on nutrition, physical activity and reducing screen time.	N=769; elementary schools, New York; 10-11 years	BMI	4% fewer boys and 2.4% fewer girls were with overweight/obesity compared with 1.3% more boys and 1.3% fewer girls in the control condition.<br>Adjusted odds ratio for boys = 0.17 (p=.04); for girls = 0.25 (p=.1)
**Haby et al. (2006) - benefits[17] & Carter et al. (2009) - costs[18]**	Multiple interventions evaluated separately:<br><br>Non-RCT; 2 years [19]	Keep your body healthy programme; nutrition and physical activity classroom teaching, parental involvement [19]<br><br>Nutrition and physical activity classroom teaching [20]	N=829; primary schools, Jerusalem; first graders (6 years) [19]<br><br>N= 5681; primary schools, Crete, Greece; first grade (6 years) [20]	BMI, dietary habits, BP, fasting total cholesterol, high density lipoproteins, triglycerides [19]<br><br>3-day food diary, BMI [20]<br><br>3-day food diary, BMI [21]	BMI residual difference between intervention and control groups = 0.76 (p<.01) [19]<br><br>1.1 units significant increase in BMI in controls compared to intervention group; no sig. difference in energy consumption [20]

	Quasi-experimental; 3 years [20]<br><br>Cluster RCT; 1 school year [21]<br><br>RCT; 2 weeks [22]†	Christchurch obesity prevention project (CHOPPS); classes discouraging fizzy drink consumption [21]<br><br>Controlling TV advertisements to moderate sugar intake and a balanced diet [22]	N=644; junior schools, Christchurch, Southwest England; 7-11 years [21]<br><br>N=288; youth camp, Quebec, Canada; 5-8 years [22]	Drink and snack choices [22]	Reduction of carbonated drink consumption in intervention group, mean difference = 0.7; 95% CI=0.1 to 0.3; decrease in overweight/obesity prevalence in intervention, mean difference = 7.7%; 95% CI = 2.2% to 13.1% [21]<br><br>Sig. effect of treatment on children's drink choice and proportion of fruit selected [22]
**Kenney et al. (2019)[23]**	An et al. (as above)	An et al. (as above)	An et al. (as above)	An et al. (as above)	An et al. (as above)
**Keszytus et al. (2013)[24]**	Cluster-randomised trial; 1 year	URMEL-ICE intervention; SSB consumption (nutrition), physical activity and media use through classroom teaching and parental engagement.	N=1810; primary schools, Ulm and Gunzburg, Germany; 7 years (average)	Parental BMI, child BMI, WC, WtHR	No statistically significant effect of intervention on BMI;<br>Unadjusted RR for incident overweight at follow-up was 0.66 (95% CI: 0.39 to 1.14) (intervention group).<br>Sig. effect on waist circumference (-0.85 (95% CI: -1.59 to -0.12).
**Kesztyus et al. (2017)[25]**	Cluster RCT; 1 year	Join the Healthy Boat intervention; SSB consumption (nutrition), physical activity and media use through classroom	N=1968; primary schools, Baden-Wurttemberg, Germany; grades 1-4	BMI, WC, WtHR.	Sig. effect of intervention on BMI percentile (mean 0.45, p = 0.038) but reduced to non-sig. when controlling for baseline BMI

		teaching and parental engagement.			
**Ladapo et al. (2016)[26]**	RCT; 5 weeks [27]	Students for Nutrition and eXercise (SNaX); promotion of healthy foods and physical activity.	N=5299; public middle schools, Los Angeles; grades 6-8	Portions of fruit and vegetables served.<br>Number of free/reduced-price lunches served.<br>Number of full price lunches served.<br>Number of all lunches served.<br>Number of snacks sold.	Increased fruit servings in intervention compared to control from pre to during intervention (0.07, (SD=0.03) p<0.01); no sig diff in veg.<br>Sig diff in snack sales (-0.03 (SD=0.01), p<0.01)
**McAuley et al. (2010)*[28]**	Non randomized Controlled intervention; 2 years [29]	A Pilot Programme for Lifestyle and Exercise (APPLE); nutrition through free fruit provision, water filters, and classroom teaching, and physical activity.	N=469; communities and primary schools, Otago, New Zealand; 5-11 years	BMI, BMI z-score, WC, Dietary intake (FFQ),<br>Physical activity	BMI z-score was significantly lower in intervention relative to control children by 0.26 units (95% CI = 0.21-0.32) at 2 years and weight z-score by 0.18 units (95% CI = 0.13-0.22).<br>Overweight (%): 0.88% increase in control relative to intervention group (95% CI: 0.69 – 1.14).
**Mernagh, Paech & Weston (2010)[30]**	McAuley et al. (as above)<br><br>Moodie et al. (as above)	APPLE (as above)<br>Be Active Eat Well (as above)<br>School Nutrition Policy Initiative (SNPI); nutrition education and school	APPLE (as above)<br>Be Active Eat Well (as above)<br><br>N=1349, primary schools,	APPLE (as above)<br>Be Active Eat Well (as above)<br><br>BMI, dietary intake (FFQ), physical activity	APPLE (as above)<br>Be Active Eat Well (as above)<br>Overweight prevalence at baseline and 2 year follow up:<br>Control: 15.89%; 20%<br>Intervention: 16.28%; 14.61%

	Cluster-RCT; 2 years [31]	policy implementation (removal of SSBs and unhealthy snacks from vending machines and cafeterias), social marketing and parent outreach. [31]	Philadelphia, USA; grades 4-6 [31]	and sedentary behaviours [31]	OR: 0.65 (95% CI: 0.54 to 0.79) P<.001<br>Overweight incidence: OR=0.67 (95% CI: 0.47 to 0.96), p=.03<br>No significant changes in obesity prevalence and incidence.<br>No sig. diff in BMI, total energy, fat and F&V consumption between groups at 2 year follow up [31]
**Moodie et al. (2013)*[32]**	Quasi-experimental non-randomized trial; 3 years [33]	Be Active Eat Well programme; nutrition (SSB, energy dense snacks and F&V), physical activity and reduction of television viewing.	N=2184; primary schools, Colac, Australia; 4-12 years	BMI, BMI z-score, WC, WtHR, self-reported physical activity and dietary intake.	Intervention group compared to control: showed lower increase in BMI scores -0.28 (95% CI: -0.7 to 0.15); had lower increases in WC -3.14 (95% CI: -5.07 to -1.22);<br>BMI Z scores -0.11 (95% CI: -0.21 to -0.01);<br>WtHR -0.02 (95% CI: -0.03 to -0.004)
**Oosterhoff et al. (2020)[34]**	Longitudinal quasi-experimental trial; 2 years[35]	Healthy Primary School of the Future (HPSF; diet and PA) vs. Physical Activity Schools (PAS; PA only); healthy morning snacks and healthy lunches,	N = 1676, n = 661 (controls), n = 537 (HPSF), n= 478 (PAS); primary schools, Netherlands; 4-12 years	BMI z-score, hip and waist circumference	Compared to control schools:<br>HPSF: − 0.21 kg/m2 [95% CI: − 0.38; −0.05]<br>PAS: − 0.17 kg/m2 [95% CI: −0.33; 0.00]<br>Effects by SES (HPSF vs controls):<br>Low SES: -0.103 (95% CI: -0.22, -0.02)

		structured sports, play and creative activities.			Middle SES: -0.049 (95% CI: -0.16; 0.06)<br>High SES: -0.063 (95% CI: -0.18; 0.05).
**Reeves et al. (2021)[36]**	Single blinded parallel group randomised controlled trial	Munch and Move (state-wide obesity prevention programme); access to a web-based menu planning and decision-support tool, online resources, online reminders and feedback.	N = 27 (intervention services), N=27 (controls services); Daycare services, New South Wales, Australia; 3-6 years.	Provision of recommended number of serves for each of the following food groups per child per day over a 1-week period:<br>(1) vegetables and legumes/beans (two serves)<br>(2) fruit (one serve)<br>(3) whole grain cereals, foods and breads (two serves)<br>(4) lean meat and poultry, fish, eggs, tofu, seeds and legumes (3/4 serve)<br>(5) milk, yoghurt, cheese and alternatives (one serve)<br>(6) no 'discretionary' foods that are high in	Mean number of guideline compliant food groups: Relative effect size 0.26; 95% CI: -0.61, 1.14; p=0.55<br>Sig. increase in servings of fruits in intervention: 0.21; 95% CI: 0.02, 0.4; p=0.03<br>Sig. reduction in mean number of times per week discretionary foods provided: -0.33; 95% CI -0.54, -0.11; p=0.003.

				energy and low in nutrients (zero serves).	
**Reilly et al. (2018)**[37]	Randomised controlled trial; 12-14 months (high intensity intervention),[38] 9 months (medium intensity intervention),[39] 12 months (low intensity intervention)[40]	Support offered in different levels of intensity to promote Government roll out of healthy canteen policy: 50% of menu to be 'green' (healthier foods), limit availability of 'amber' and restrict sale of 'red' (poor nutritional value). Sugar sweetened beverage sale ban in schools.<br><br>High intensity: support, provision of tools, performance monitoring and feedback, bi-monthly school visits.<br><br>Medium intensity: **:** as high-intensity but text-message based	High intensity: N=35 (intervention), N=35 (control).<br><br>Medium intensity: N=28 (intervention), N=25 (control).<br><br>Low intensity: N=36(intervention), N=36 (control).<br><br>Primary Schools, New South Wales, Australia, 5-12 years.	Proportion of canteen menus that (i) did not contain items restricted for sale (red/banned); (ii) healthy canteen items (green) represented more than 50% of listed menu items.	Policy adherent school canteen menu, compared to controls:<br>High intensity: RR=14.41; 95% CI: 2.08, 99.97; p=<0.001<br>Medium intensity: RR=4.29; 95% CI: 1.04, 17.68; p=0.02<br>Low intensity: RR=4.44; 95% CI: 0.65, 30.11; p=0.06

		support. Two support contacts per school term.<br><br>Low intensity: canteen menu audits with provision of feedback via written report or telephone call each school term (4x).			
**Rush et al. (2014)[41]**	Longitudinal Randomized controlled trial; 2 years [42,43]	Project Energize; healthy eating and physical activity.	N=192 intervention schools (124 control schools (2004) and 62 schools (2006); primary schools, Waikato District, New Zealand; 6-8 years (“younger children”) and 9-11 years (“older children”)	BMI, WC, BP, Fitness<br>Body composition<br>Healthy eating habits and physical activity questionnaire	2006 control data comparison: median BMI difference 0.504 kg/m2 (90% CI: -0.435 to -0.663)<br>2004 BMI comparison: -0.551 kg/m2 (90% CI: -0.456 to -0.789)
**Te Velde et al. (2011)[44]**	Cluster randomized trial; 2 years [45,46]	Pro children; provision of healthy foods, curriculum activities, parental involvement [46]	N=735[46]<br>N=771; [45] primary schools, Netherlands; 5th grade (10 years)	F&V consumption	Intervention group consumed 28.7g/day more F&V than control (95% CI= -12.8;70.1).[46]<br>Intervention group consumed 17.4g/day more F&V than control (95% CI= -0.9;35.6).[45]

		Schoolgruiten; free fruit/veg, curriculum-based knowledge and skill development[45]			Neither statistically significant.
**Vieira et al. (2019)[47]**	Non-randomised controlled trial	Planning Health in School Programme; 8x45 minute learning modules to improve diet and F&V intake, increase PA and reduce TV viewing; 10 months.	N=219 (intervention), N=230 (controls); primary schools, Trofa municipality, Porto, Portugal; 10-14 years	Height, weight, WC, BMI, WHtR, FFQ	BMI: Intervention (mean=0.12, SD=0.94) vs control (mean=0.21, SD=1.01); p=0.35<br>WC: Intervention (mean=-0.38, SD=2.81) vs control (mean=0.3, SD=2.98); p=0.015<br>WHtR: Intervention (mean=-0.01, SD=0.02) vs control (mean=-0.008, SD=0.02); p=0.002<br>Less soft drink consumption from 0.7 to 0.5 servings/day in intervention group (p=0.043)
**Wang et al. (2008)[48]**	RCT; 3 years [49]	FitKid project; after school programme, healthy snacking, physical activity, discouraging sedentary behaviours.	N=890; elementary schools, Augusta, Georgia, USA; 3rd graders	Reduction in %BF, BMI, WC	At least 40% of after school sessions reduced %BF by 0.76% (95% CI: -1.42 to -0.09) compared with control.
**Wang et al. (2003)[50]**	Randomized controlled trial; 2 years [51]	Planet Health; nutrition, physical activity and television viewing, incorporated within	N=1560; middle schools, Boston, Massachusetts; grades 6-7	BMI and tricep-skinfold	Intervention: Obesity prevalence declined from 23.6% to 20.4% during the two-year intervention.<br>Controls: Obesity prevalence increased from 21.5% to 23.7%.

		interdisciplinary curriculum.			Obesity prevalence sig. reduced in intervention girls compared to controls (OR=0.47, 95% CI: 0.24 to 0.93, p=0.03).
**Wyatt et al. (2018)[52]**	Cluster RCT; 3 school terms	Healthy Lifestyles Programme (HeLP); SSB consumption, healthy and unhealthy snacks, physical activity and screen time, classroom teaching and activities.	N=1324; state primary and junior schools, Devon, UK; year 5 students	BMI-z at 24 months (primary)<br>BMI-z at 18 months, WC-z, %BF-z, % children classified as underweight/healthy weight/overweight/obese	BMI-z at 24months: -0.02 (95% CI: -0.09 to 0.05; p=0.567)<br>WC-z: -0.05 (95% CI: -0.23 to 0.13)<br>%BF: -0.03 (95% CI: -0.61 to 0.55)

Abbreviations: BF, body fat; BMI, Body Mass Index; BP, blood pressure; CHU9D, Child Health Utility Index 9-dimensions; CI, Confidence intervals; FFQ, food frequency questionnaire; F&V, Fruit and Veg; HPSF, healthy primary school of the future; kilojoule, kJ; kcal, kilocalorie; OR, odds ratio; PA, physical activity; PAS, physical activity school; RCT, randomised controlled trial; RR, relative risk; SES, socioeconomic status; SSB, sugar sweetened beverages; WC, waist circumference; WtHR, waist to height ratio

*intervention based in community and school setting

†intervention based in youth camp setting

References

1. Adab P, Barrett T, Bhopal R, et al. The West Midlands ActiVe lifestyle and healthy Eating in School children (WAVES) study: a cluster randomised controlled trial testing the clinical effectiveness and cost-effectiveness of a multifaceted obesity prevention intervention programme targeted at children aged 6-7 years. *Health Technol Assess.* 2018;22(8):1-608.
2. An R, Xue H, Wang L, Wang Y. Projecting the impact of a nationwide school plain water access intervention on childhood obesity: a cost-benefit analysis. *Pediatr Obes.* 2018;13(11):715-723.
3. Schwartz AE, Leardo M, Aneja S, Elbel B. Effect of a school-based water intervention on child body mass index and obesity. *JAMA Pediatr.* 2016;170(3):220-226.
4. Beets MW, Brazendale K, Weaver R, et al. Economic evaluation of a group randomized controlled trial on healthy eating and physical activity in afterschool programs. *Prev Med: Int J Devoted to Pract and Theory.* 2018;106:60-65.
5. Beets MW, Weaver RG, Turner-McGrievy G, et al. Two-year healthy eating outcomes: an RCT in afterschool programs. *Am J Prev Med.* 2017;53(3):316-326.
6. Brown IHS, Perez A, Li YP, Hoelscher DM, Kelder SH, Rivera R. The cost-effectiveness of a school-based overweight program. *Int J Behav Nutr Phys Act.* 2007;4(47).
7. Coleman KJ, Tiller CL, Sanchez J, et al. Prevention of the epidemic increase in child risk of overweight in low-income schools: the El Paso coordinated approach to child health. *Arch Pediatr Adolesc Med.* 2005;159(3):217-224.
8. Brown A, Sutherland R, Reeves P, Nathan N, Wolfenden L. Cost and Cost Effectiveness of a Pilot m-Health Intervention Targeting Parents of School-Aged Children to Improve the Nutritional Quality of Foods Packed in the Lunchbox. *Nutrients.* 2021;13(11).
9. Sutherland R, Nathan N, Brown A, et al. A randomized controlled trial to assess the potential efficacy, feasibility and acceptability of an m-health intervention targeting parents of school aged children to improve the nutritional quality of foods packed in the lunchbox 'SWAP IT'. *Int J Behav Nutr Phys Act.* 2019;16(1):54.

10. Coffield E, Nihiser A, Carlson S, et al. Shape Up Somerville's return on investment: Multi-group exposure generates net-benefits in a child obesity intervention. *Prev Med Rep.* 2019;16:100954.
11. Economos CD, Hyatt RR, Must A, et al. Shape Up Somerville two-year results: a community-based environmental change intervention sustains weight reduction in children. *Prev Med.* 2013;57(4):322-327.
12. Conesa M, Llaurado E, Aceves-Martins M, et al. Cost-Effectiveness of the EdAl (Educacio en Alimentacio) Program: A Primary School-Based Study to Prevent Childhood Obesity. *J Epidemiol.* 2018;28(12):477-481.
13. Tarro L, Llauradó E, Albaladejo R, et al. A primary-school-based study to reduce the prevalence of childhood obesity–the EdAl (Educació en Alimentació) study: a randomized controlled trial. *Trials.* 2014;15(1):58.
14. Ekwaru JP, Ohinmaa A, Tran BX, Setayeshgar S, Johnson JA, Veugelers PJ. Cost-effectiveness of a school-based health promotion program in Canada: A life-course modeling approach. *PLoS ONE.* 2017;12(5):e0177848.
15. Fung C, Kuhle S, Lu C, et al. From" best practice" to" next practice": the effectiveness of school-based health promotion in improving healthy eating and physical activity and preventing childhood obesity. *Int J Behav Nutr Phys Act.* 2012;9(1):27.
16. Graziose MM, Koch PA, Wang YC, Gray HL, Contento IR. Cost-effectiveness of a Nutrition Education Curriculum Intervention in Elementary Schools. *J Nutr Educ Behav.* 2017;49(8):684-691.
17. Haby M, Vos T, Carter R, et al. A new approach to assessing the health benefit from obesity interventions in children and adolescents: The assessing cost-effectiveness in obesity project. *Int J Obes.* 2006;30(10):1463-1475.
18. Carter R, Moodie M, Markwick A, et al. Assessing cost-effectiveness in obesity (ACE-obesity): an overview of the ACE approach, economic methods and cost results. *BMC Public Health.* 2009;9:419.
19. Tamir D, Feurstein A, Brunner S, Halfon S-T, Reshef A, Palti H. Primary prevention of cardiovascular diseases in childhood: changes in serum total cholesterol, high density lipoprotein, and body mass index after 2 years of intervention in Jerusalem schoolchildren age 7–9 years. *Prev Med.* 1990;19(1):22-30.

20. Manios Y, Moschandreas J, Hatzis C, Kafatos A. Evaluation of a health and nutrition education program in primary school children of Crete over a three-year period. *Prev Med.* 1999;28(2):149-159.
21. James J, Thomas P, Cavan D, Kerr D. Preventing childhood obesity by reducing consumption of carbonated drinks: cluster randomised controlled trial. *BMJ.* 2004;328(7450):1237.
22. Gorn GJ, Goldberg ME. Behavioral evidence of the effects of televised food messages on children. *J Consum Res.* 1982;9(2):200-205.
23. Kenney EL, Cradock AL, Barrett JL, et al. Cost-Effectiveness of Water Promotion Strategies in Schools for Preventing Childhood Obesity and Increasing Water Intake. *Obesity.* 2019;27(12):2037-2045.
24. Kesztyus D, Schreiber A, Wirt T, et al. Economic evaluation of URMEL-ICE, a school-based overweight prevention programme comprising metabolism, exercise and lifestyle intervention in children. *Eur J Health Econ.* 2013;14(2):185-195.
25. Kesztyüs D, Lauer R, Kesztyüs T, Kilian R, Steinacker JM. Costs and effects of a state-wide health promotion program in primary schools in Germany - the Baden-Württemberg Study: a cluster-randomized, controlled trial. *PLos ONE.* 2017;12(2):e0172332.
26. Ladapo JA, Bogart LM, Klein DJ, et al. Cost and Cost-Effectiveness of Students for Nutrition and eXercise (SNaX). *Acad Pediatr.* 2016;16(3):247-253.
27. Bogart LM, Cowgill BO, Elliott MN, et al. A randomized controlled trial of students for nutrition and eXercise: a community-based participatory research study. *J Adolesc Health.* 2014;55(3):415-422.
28. McAuley KA, Taylor RW, Farmer VL, et al. Economic evaluation of a community-based obesity prevention program in children: the APPLE project. *Obesity (Silver Spring).* 2010;18(1):131-136.
29. Taylor RW, McAuley KA, Williams SM, Barbezat W, Nielsen G, Mann JI. Reducing weight gain in children through enhancing physical activity and nutrition: the APPLE project. *Int J Pediatr Obes.* 2006;1(3):146-152.
30. Mernagh P, Paech A, Coleman K, et al. Assessing the cost-effectiveness of public health interventions to prevent obesity: overview report. Wellington: Health Research Council of New Zealand. 2010.
31. Foster GD, Sherman S, Borradaile KE, et al. A policy-based school intervention to prevent overweight and obesity. *Pediatr.* 2008;121(4):e794-e802.

32. Moodie ML, Herbert JK, de Silva-Sanigorski AM, et al. The cost-effectiveness of a successful community-based obesity prevention program: the be active eat well program. *Obesity (Silver Spring).* 2013;21(10):2072-2080.
33. Sanigorski AM, Bell AC, Kremer PJ, Cuttler R, Swinburn BA. Reducing unhealthy weight gain in children through community capacity-building: results of a quasi-experimental intervention program, Be Active Eat Well. *Int J Obes (Lond).* 2008;32(7):1060-1067.
34. Oosterhoff M, Over EAB, van Giessen A, et al. Lifetime cost-effectiveness and equity impacts of the Healthy Primary School of the Future initiative. *BMC Public Health.* 2020;20(1):1887.
35. Bartelink NHM, van Assema P, Kremers SPJ, et al. Can the Healthy Primary School of the Future offer perspective in the ongoing obesity epidemic in young children? A Dutch quasi-experimental study. *BMJ Open.* 2019;9(10):e030676.
36. Reeves P, Edmunds K, Szewczyk Z, et al. Economic evaluation of a web-based menu planning intervention to improve childcare service adherence with dietary guidelines. *Implement Sci.* 2021;16(1):1.
37. Reilly KL, Reeves P, Deeming S, et al. Economic analysis of three interventions of different intensity in improving school implementation of a government healthy canteen policy in Australia: costs, incremental and relative cost effectiveness. *BMC Public Health.* 2018;18(1):378.
38. Wolfenden L, Nathan N, Janssen LM, et al. Multi-strategic intervention to enhance implementation of healthy canteen policy: a randomised controlled trial. *Implement Sci.* 2017;12(1):6.
39. Nathan N, Yoong SL, Sutherland R, et al. Effectiveness of a multicomponent intervention to enhance implementation of a healthy canteen policy in Australian primary schools: a randomised controlled trial. *Int J Behav Nutr Phys Act.* 2016;13(1):106.
40. Yoong SL, Nathan N, Wolfenden L, et al. CAFE: a multicomponent audit and feedback intervention to improve implementation of healthy food policy in primary school canteens: a randomised controlled trial. *Int J Behav Nutr Phys Act.* 2016;13(1):126.
41. Rush E, Obolonkin V, McLennan S, et al. Lifetime cost effectiveness of a through-school nutrition and physical programme: Project Energize. *Obes Res Clin Pract.* 2014;8(2):e115-e122.

42. Rush E, Graham D, McLennan S, Latimer K. An evaluation of nutrition and physical activity in Waikato primary schools (Project Energize: June 2008 to June 2011). Hamilton: Waikato District Health Board and Ministry of Health, Healthy Eating Healthy Action Evaluation Fund. 2011.
43. Rush E, Reed P, McLennan S, Coppinger T, Simmons D, Graham D. A school-based obesity control programme: Project Energize. Two-year outcomes. *Br J Nutr.* 2012;107(4):581-587.
44. Te Velde SJ, Lennert Veerman J, Tak NI, Bosmans JE, Klepp KI, Brug J. Modeling the long term health outcomes and cost-effectiveness of two interventions promoting fruit and vegetable intake among schoolchildren. *Econ Hum Biol.* 2011;9(1):14-22.
45. Tak NI, te Velde SJ, Brug J. Long-term effects of the Dutch Schoolgruiten Project–promoting fruit and vegetable consumption among primary-school children. *Public Health Nutr.* 2009;12(8):1213-1223.
46. Te Velde S, Brug J, Wind M, et al. Effects of a comprehensive fruit-and vegetable-promoting school-based intervention in three European countries: the Pro Children Study. *Br J Nutr.* 2008;99(4):893-903.
47. Vieira M, Carvalho GS. Costs and benefits of a school-based health intervention in Portugal. *Health Promot Int.* 2019;34(6):1141-1148.
48. Wang LY, Gutin B, Barbeau P, et al. Cost-effectiveness of a school-based obesity prevention program. *J School Health.* 2008;78(12):619-624.
49. Yin Z, Gutin B, Johnson MH, et al. An environmental approach to obesity prevention in children: Medical College of Georgia FitKid Project year 1 results. *Obes Res.* 2005;13(12):2153-2161.
50. Wang LY, Yang Q, Lowry R, Wechsler H. Economic analysis of a school-based obesity prevention program. *Obes Res.* 2003;11(11):1313-1324.
51. Gortmaker SL, Peterson K, Wiecha J, et al. Reducing obesity via a school-based interdisciplinary intervention among youth: Planet Health. *Arch Pediatr Adolesc Med.* 1999;153(4):409-418.
52. Wyatt K, Lloyd J, Creanor S, et al. Cluster randomised controlled trial and economic and process evaluation to determine the effectiveness and cost effectiveness of a novel intervention [Healthy Lifestyles Programme (HeLP)] to prevent obesity in school children. *Public Health Res.* 2018;6(1).

TABLE S5 Long-term modelling methods of cost and benefit outcomes in economic modelling studies

Study	Methods	Databases utilised
**An et al. (2018)**	Each Markov cycle exposed subjects to an age and sex-specific risk of death. Survivors gain a year of life and corresponding costs should they be with overweight/obesity. Simulation ends when all subjects die.<br>Probability parameters of an overweight child to become an adult with overweight/obesity were obtained from the literature.<br>The model assumed that economic costs of overweight/obesity begin to accumulate from age 35.<br><br>Normal distributions were assigned to all parameters.	Age and sex specific risk of death parameters obtained from the United States Life Tables, 2011.<br><br>Nationally representative health survey data (Finkelstein et al.,; Dor et al.,; Tsai et al.) obtained per capita annual medical costs associated with adult overweight and obesity. Adjusted for inflation based on the Consumer Price Index issued by the US Bureau of Labor Statistics.<br><br>National Health and Nutrition Examination Survey – prevalence of adult overweight/obesity.<br>p<br>National Centre for Education Statistics – total number of public and private schools in the United States, including 2015 enrolment figures.
**Brown et al. (2007)**	**Intervention outcomes:** childhood obesity cases averted based on obesity status at 11 years → predict obesity cases averted at 25-29 years → predict obesity cases averted at 40-64 → include intervention costs → include medical costs averted at 40-64 years (and estimated labour productivity costs averted) based on obesity cases averted → estimate QALYs and calculate cost-effectiveness ratio (CER), or estimate net benefit.	Lifetime obesity progression model: probabilities estimated by linking **1992, 1987, 1982 NHANES I Epidemiological Follow up Study (NHEFS)** data with the original **1975 National Health and Nutrition Examination Survey (NHANES) I** data.

	Poisson regression was used to estimate number of lost sick days for individuals with and without obesity. Life expectancy and mortality by gender was calculated for 40 year olds with and without obesity who died before turning 65.<br>U.S. Department of Labour, Bureau of Labor Statistics Population Survey Data was used to place value on sick days averted.<br><br>**Lifetime obesity progression model** - predicted adulthood obesity based on child overweight. This model requires the following information: number of participants at follow up; proportion of at-risk/overweight in grades three and five in the control and intervention arms separately; probability of obesity at 21-29 years conditional on being at-risk, with obesity, not at-risk, without obesity at 11 years; probability of obesity at 40 years conditional on being with obesity and without obesity at 20-29 years.	QALYs = **2002 NHIS survey questions** on self-reported health and activity limitations.<br><br>Life tables by Peeters et al. (2003) used to project life expectancy at 40.<br><br>**Medical cost parameters:** NHANES III - estimate costs for hyper-tension, hypercholesterolemia, type 2 diabetes, cardiovascular disease and stroke covering age period of 35 years - death.<br><br>**Indirect costs** calculated using 2002 National Health Interview Survey data.
**Coffield et al. (2019)**	Capital expenditures were annuitized at a 3% rate with a 10 year lifespan, and future benefits were discounted at an annual 3% rate. Costs were adjusted to 2014 USD using either the Center for Medicare Studies' Health Care Expenditure Price Index or the Consumer Price Index.<br>Intervention effect size depreciated by 2.62% annually (calculated based on estimation of a "breakeven" depreciation rate where costs remain equal to the program's estimated benefits).<br>Healthcare cost estimation: based on changes in BMI z-score (children) or BMI (parents) changes using the Medical Expenditure Panel Surveys (MEPS). Twenty age-specific samples were created to reflect estimated costs by age (child) or age-group (parent) over the 10-year horizon. Regressions tested for significant associations between healthcare costs and BMI changes at each age	Center for Medicare Studies Health Care Expenditure Price Index (healthcare cost adjustments only)

	sample controlling for socioeconomic and demographic covariates. Healthcare costs averted were only considered for significant associations.<br>Productivity loss averted were estimated annually for parents only, based on number of sickness-related missed workdays associated with a 1-point BMI change; parent population-wide treatment effect; and median wage estimates of the MEPS sample.	
**Ekwaru et al. (2017)**	Markov model based on 10 cohorts of students who pass through grade 5 over a ten-year period.<br>The model assumed that pupils' body weight status predicted their adult body weight, which determined their risk of weight-related diseases and quality of life.<br>It was assumed that the lifestyle changes developed in the two intervention years would continue on for 8 more years.<br>The model included 43 states - three weight categories (normal weight, overweight, obese) and 13 chronic diseases with links to weight status, no-chronic disease state and the dead state. Disease states included: diabetes, hypertension, asthma, osteoarthritis, stroke, coronary heart disease (CHD), kidney cancer, pancreatic cancer, colorectal cancer, breast cancer, endometrial cancer, ovarian cancer, and gallbladder cancer).<br>A multinomial logistic regression model was used to fit data from these cohorts, based on 3 independent variables (sex, weight status and age at a given time point), and outcome variables (weight status in the next assessment (every 2 years). Parameter estimates of the fitted multinomial logistic regression were used in the Markov model to obtain sex and age specific weight status transition probabilities.<br><br>Conditional probability of dying given weight and chronic disease status: Canadian Life Table used to extract mortality hazards by sex and age based on	**Weight status transition probabilities**: National Population Health Survey (NPHS) (follows 12 year old children and older); National Longitudinal Survey of Children and Youth (NLSCY) that follows children under 12 years of age.<br><br>**Probabilities of developing chronic diseases**: published studies on incidence rates of chronic diseases, effects of weight status on incidence rates (Guh et al., 2009) and weight status distributions from Statistics Canada 2010.<br><br>Mortality probabilities: Canadian life table.

	the general Canadian Population, as well as the effects of weight status and chronic disease on all-cause mortality (weight status distribution and prevalence of chronic diseases were considered).<br>At each cycle (age), mortality hazard was estimated based on sex, age and weight status (Mortality hazard obtained from life tables X (Relative Risk of dying for weight status given sex and age / the sum of the proportion of people in a particular weight status given their sex and age multiplied by the relative risk of dying for a particular weight status given sex and age).<br><br>For every year lived with excess weight/obesity/chronic disease, there was an assigned decrement in health utility scores.	
**Graziose et al. (2017)**	Quantification of benefits based on QALYs.<br>A hypothetical model population of fifth grade students with a distribution of observable demographic characteristics and weight status was taken from publicly available data.<br>Trial effectiveness data was used to estimate reduction in obesity at age 10 years for males and female. Childhood-to-adulthood BMI trajectories were used to estimate adult obesity prevalence.<br>QALYs were estimated based on existing data taken from Muennig et al.<br>Medical costs averted were obtained from Finkelstein et al.<br>A CER was calculated to include medical costs associated with obesity averted.	**Obesity progression model** used to estimate number of cases of adult obesity averted due to the intervention.<br><br>**Childhood-to-adulthood BMI trajectories** from Goldhaber-Robert et al (2013).
**Haby et al. (2006) benefits &**	Where interventions have gathered behavioural outcomes rather than anthropometric outcomes, the relationship between behaviour change, energy balance and BMI was modelled:<br>1) determine changes in energy consumed from behaviour change.<br>2) model impact on changes in child weight – 10% change in energy balance resulted in 4.5% change in body weight (95% CI= 3.8; 5.1).	**Australian 1995 National Survey** (NNS95) – 5-19 y/o used as cohort of children for the model; weight and energy density of total diet was used to determine energy imbalance from behaviour change; mean changes in weight

**Carter et al. (2009) - costs**	3) model DALYs saved as a difference in mortality and morbidity outcomes, in 5 year increments, by sex, due to intervention effects which may result to changes in age-specific BMI distribution over the lifetime, in comparison to a control, through the use of life tables.<br>4) calculated Potential Impact Fraction (PIF) which is the proportional change in expected disease/death attributable to intervention/control condition. This is used to determine the impact of change in BMI distribution of mortality and morbidity (9 diseases considered). Relative risk estimates for 30-44 year olds were also applied to the 25-29 age group.<br>5) Reduction in obesity related costs were calculated using the same methods as DALYs saved.	were translated to mean changes in BMI, assuming constant height.<br><br>**Victorian Burden of Disease Study** – years of life lived for disease-related disability.<br><br>**Australian Institute of Health and Welfare** – used to calculate cost offsets.
**Kenney et al. (2019)**	CHOICES microsimulation model was used to estimate costs, population reach, water intake, health outcomes and health care cost savings related to childhood obesity over a 10-year time horizon from 2015 to 2025. The model simulates individuals in the US population to project how children's individual growth trajectories would shift after exposure to the intervention, and how that would impact on health and health care costs. Growth trajectories were estimated based on data on demographic characteristics, growth, health behaviours and obesity risk from multiple national datasets. The model was used to estimate the expected reductions in BMI and number of cases of childhood obesity prevented after 10 years. Annual health care cost savings were estimated based on published estimates of healthcare costs associated with child and adult obesity.	Labour costs – Bureau of Labor Statistics (45.56% fringe rate)<br>US Census;<br>The American Community Survey;<br>The National Survey of Children's Health<br>The National Health and Nutrition Examination Survey;<br>The Early Childhood Longitudinal Study-kindergarten cohort;<br>The Behavioral Risk Factor Surveillance system
**Mernagh, Paech & Weston (2010)**	Control arm of economic model: simulation to estimate BMI of 10,000 individuals for each ethnic group, for each age between 2-75 years. Each individual was categorised as healthy weight, with overweight or with obesity. The impact of the intervention on mean BMI was subtracted for each simulated individual → this produces a new intervention cohort of 10,000	**New Zealand life tables** – mortality estimated in each yearly cycle.<br><br>**Statistics New Zealand (2009) National Population Estimates** – mortality rate data

	individuals. Probabilities (expected incidence) of staying in good health or contracting one of 14 obesity-related chronic illnesses was applied to the model at each yearly control group cycle, by age (using Dutch data due to unavailability of New Zealand data).<br>Incidence estimates applied to those 20 years+.<br>Utility weights (sourced from the literature) were applied to health states representing average quality of life over the duration of the illness. Utility weights were not applied to BMI categories.<br>Intervention effects were applied to a five-year timeframe (where follow up was not that long, the last follow up effect size was carried forward).<br><br>Reduction in BMI (intervention effect) relative to controls would decay by 1% per annum after 5 years within the economic model.	**Mittman et al. (1999)** – utility weights.<br><br>**New Zealand Ministry of Health (2004)** – annual health care costs by age and gender – on-going health care costs for healthy weight individuals.<br><br>**Baal et al. (2008)** – cost of illness data for chronic disease. Ratio applied to estimate incremental cost of chronic illness for New Zealand population.
**Moodie et al. (2013)**	**BMI to DALYs**:<br>Reduction in BMI was converted to DALYs saved using the ACE-Obesity model. DALYs averted were calculated as the difference in future morbidity and mortality between intervention and control groups.<br>PIFs were used to calculate the impact of the change in BMI on expected disease or death.<br>Diseases considered in the model: ischaemic heart disease, ischaemic stroke, hypertensive heart disease, type 2 diabetes, osteoarthritis, endometrial cancer, colon cancer, postmenopausal breast cancer and kidney cancer.<br>The Markov Model takes the cohort of children (aged 5-19) and follows them in five-year increments in separate gender groups, until 100 years of age (or death).	2001 population epidemiology and disease cost data.

	The intervention was modelled at a national level for one year. It was assumed that the intervention would be taken up by 10% of Australian Primary Schools.	
**Oosterhoff et al. (2020)**	Modelling started from age 4 – lifetime (distinguished between childhood/adolescence 4-20 years and adulthood). Lifetime health and cost impacts were modelled through changes in BMI.<br>Intervention effects were relayed onto a BMI trajectory (extrapolated BMI values up to age 20 years).<br>School day extended to 30 mins per day, 4 days a week.<br>School absenteeism considered as productivity indicator for children. (excess missed school days associated with overweight/obesity obtained from the literature).<br>HRQoL weights and healthcare costs (GP and specialist visits) by child weight status obtained from literature.<br>Costs and QALYs calculated for each age cohort between 4-12 years and then aggregated to represent Dutch 4-12 year olds in a school cohort. RIVM Chronic Disease Model was used to project effects from 20 years of age up to 100 (lifetime). Markov model approach; prevalence, incidence and mortality of chronic diseases based on changes in risk factors and weight category (normal weight, overweight and obesity). Considers diseases during life years gained. Diseases included: myocardial infarction (AMI), angina pectoris, chronic heart failure, stroke, renal, colorectal, breast, prostate, and endometrium cancer, diabetes mellitus, hip and knee arthritis, and low back pain. The model also considers the risk of secondary diseases due to primary diseases (independent of weight). Model also considers diseases due to ageing (independent of weight). This included: chronic obstructive pulmonary disease (COPD), lung, stomach, esophagus, larynx, bladder, pancreas, and oral cavity cancer.	**Lehnert et al. (2014)** - Productivity losses by weight category<br><br>**Dutch Burden of Disease Study (Melse et al., 2000)** - Adult Utility weights<br><br>Child utility weights – literature<br><br>**Dutch Cost of Illness Study (Slobbe et al., 2006)** - Health resource use and costs:<br><br>**Zorginstituut Nederland (2015)** - Productivity and healthcare costs

	Presence of disease determined utilities obtained from the Dutch Burden of Disease Study. Health state utilities for overweight and obesity are not included. Gains in HRQoL is based on decreases in prevalence of disease and not from weight loss. Healthcare costs were estimated based on the Dutch Cost of Illness Study.<br>Productivity losses – relation between weight category in adulthood and number of annual sick leave days from work, calculated up to 67 years of age (retirement).<br>Equity efficiency impact plane, displaying trade-offs between cost-effectiveness and health equity.<br>Controls: Assumed BMI changes only due to growth.	
**Rush et al. (2014)**	The economic model was adapted from Health Research Council of New Zealand (2010).<br>Intervention costs were offset against life-time obesity health treatment costs averted.<br>The model estimates QALY increase given obesity-related health conditions averted.<br><br>Intervention costs and effects were extrapolated for a lifetime → these were translated onto New Zealand population BMI distributions. In each year of age (2-75), the population is categorised as either normal weight, with overweight or with obesity by fitting a lognormal distribution to mean BMI and standard errors using population survey data.<br><br>The model tracks risk and projects prevalence of 14 obesity-related diseases and full health, along with associated costs and health benefits (life years and preference-based utility weights).	New Zealand Ministry of Health 2006/2007 population survey data – used to obtain population BMI data.<br><br>New Zealand life tables and Dutch data on relative risks of disease incidence and mortality conditional on BMI.

	Each health state is associated with a preference-based utility.<br>An intervention is modelled by how it shifts the distribution of BMI in both males and females in the general and Maori populations, using data from intervention effects on BMI.<br><br>A 1% decay in intervention effects was applied after first 5 years of the intervention.<br>The intervention was modelled by applying observed change in median BMI to the relevant comparison cohort for both general and Maori populations.	
**Te Velde et al. (2011)**	Epidemiological modelling was used to estimate future health effects of increased fruit and veg intake.<br>The modelling procedure involved, 1) estimation of the effect of the intervention on fruit and veg consumption at 2 years; 2) translating consumption effects into changes in health outcomes → the model compares a reference population (general Dutch population) to an identical intervention population where the amount of fruit and veg consumed can be changed (according to the intervention effects). Mean intake of F&V (g/day) were fit to a Gamma distribution (higher mean for intervention conditions). Intervention consumptions levels at follow up were extrapolated over a lifetime using 30% of intervention effects to track effects from young adulthood to late adulthood.<br><br>Disease incidence due to F&V intake was quantified via PIFs (changes in incidence due to changes in exposure) using a given formula. Disease incidence in the intervention population was calculated from PIFs and incidence in general population.<br>The model did not account for incidence and mortality rates from causes other than the diseases included. The model accounts for incidence and mortality in	**National Food Consumption Survey 2003** – provides F&V consumption data for the Dutch reference population covering ages 19-30 years.<br><br>**WHO Comparative Risk Assessment** exercise – relative risk estimates.<br><br>**GP registries** – incidence data<br><br>**Netherlands National Institute for Public Health and Environment 2000** – incidence and mortality estimates for general population.<br><br>**DisMod II tool** – enforces consistency between different epidemiological data.<br><br>**Dutch disability weights** – used to estimate HRQoL lost due to disease (weights are based

	the intervention population separately for men and women, whereby life expectancy and disability-adjusted life expectancy is calculated.<br><br>Incremental cost-effectiveness ratio (ICER) and net-monetary benefit (NMB) was calculated with and without inclusion of lifetime healthcare costs.<br><br>Each intervention was compared to a "No intervention" scenario, as well as against each other.	on severity levels, therefore used estimates of the distribution by the National Institute for Public Health and the Environment to get average disability weight for each disease).<br><br>Costs of disease (Slobbe et al., 2006)
**Wang et al. (2003)**	CER was calculated as a ratio of net intervention costs to total QALYs saved by the intervention.<br>Net benefit was calculated as costs averted by the intervention minus intervention costs.<br>Analysis was undertaken for females only as no significant reduction in prevalence of overweight was found amongst boys.<br><br>Base case analysis:<br>1) estimation of intervention costs;<br>2) translating observed overweight reduction at 14 years onto overweight prevented at 40 years through the development of a two-stage overweight progression model;<br>3) estimation of medical care costs averted, QALYs saved, and productivity costs averted, per case of adulthood overweight prevented;<br>4) calculation of cost effectiveness ratio and net benefit of the intervention.<br><br>**Overweight progression model (decision tree):** Students were separated into groups with and without obesity for the intervention and a hypothetical no intervention condition, at 14 years. They were further classified as overweight or not at 40 years. By comparing expected number of adulthood overweight	**Cases of adulthood overweight prevented**: Estimates taken from Whitaker et al. (1997) to predict overweight in 21-29 year olds from 1-17 year olds.<br>**NHANES I Epidemiological Follow-up Study** (EFS) - probability of 21-29 year old women with a BMI >27.3 kg/m2 becoming overweight by 40 years (defined as BMI >25kg/m2) and probability of non-overweight 21-29 year old becoming overweight by age 40.<br><br>**Medical costs averted:** incidence-based analysis from Gorsky et al. (1996) – direct health care and medication costs associated with women at 40 years and maintained overweight to age 65 years.<br><br>**QALYs saved**: Healthy People 2000 years of healthy life (YHL) measure (developed by

	cases by age 40 between the two conditions, an estimation of overweight cases prevented by the intervention was calculated.<br><br>**Medical costs averted in years 40-65 years for the following conditions**: Coronary heart disease, hypertension, diabetes, symptomatic gallstones, and osteoarthritis.<br>Medical costs averted = $4132 ($2229 moderately overweight - $5325 severely overweight).<br><br>**QALYs saved per case of adulthood overweight prevented**: calculated mean years of healthy life (YHL) scores by BMI and combined these with the life expectancy estimates, through linear regression techniques, to calculate QALYs for overweight and non-overweight women.	National Centre for Health Statistics) and 1990 National Health Interview Survey (NHIS).<br><br>**Costs of lost productivity**: 1990 NHIS of the Health Promotion and Disease Prevention sample person file used to estimate average work absenteeism in 40-64 year old women by BMI status.<br><br>**Bureau of Labour Statistics** used to calculate median weekly earnings of the nation in 1996. 35-54 year old women, median earnings = $468 per week; $93.6 a day, and $25272 per year.
**Wyatt et al. (2018)**	Exeter Obesity Model – a two stage economic model: predicted adult weight status from participant weight status at follow up (age 11-12 years); then predicted future weight-related health outcomes as a consequence of predicted adult weight, through a Markov model approach.<br><br>Weight-related health outcomes included were type 2 diabetes, chronic heart disease, stroke and colorectal cancer.<br><br>Each model cycle was 1 year. Adults entered the model as either healthy weight, with overweight or with obesity (disease free). At each cycle year, adults have a probability of either remaining in an event-free state, develop a weight-related disease state, or death. Each cycle comes with an annual mortality risk for event-free and disease-specific mortality for disease states.	**Unit Costs for Health and Social Care** – unit costs for staff inputs<br><br>**Power et al. 1997** – UK longitudinal study tracking 7 year olds until 33 years; used to predict adult weight status from intervention outcomes.<br><br>**UK Office for National Statistics** – all-cause mortality risk<br><br>**Health State values to derive QALYs** obtained from a literature search.

Costs for disease states (treatment costs) were applied and inflated/uprated to 2014/2015 where necessary.

TABLE S6 Adjusted parameters within sensitivity analysis	
**Parameter**	**Examples**
Intervention implementation costs	• Price substitutions using different data sources [1]<br>• Increasing or decreasing costs by a set percentage [2-5]<br>• Comparator costs varied [6]<br>• Teacher wages varied to test intervention implementation at different locations [7]<br>• Salary costs [8,9]<br>• Inclusion of sunk costs (one-off bulk cost for intervention development) [5,10,11]<br>• 95% confidence intervals associated with costs used to get lower and upper bounds of economic analysis estimates.[12,13]
Opportunity costs	• Included opportunity costs of time taken from parents (e.g. work days lost) [7,10]<br>• Ratio of school absenteeism/sick leave days days for overweight and obesity vs. normal weight varied +/- 20%[5]
Medical costs	• Medical costs obtained from different source [7,14,15]<br>• Ratio of healthcare costs for overweight and obesity vs. normal weight varied +/- 20%[5]
Intervention effectiveness parameters [4,5,8,13,16-21]	• Influence of intervention effect (BMI) rate of decay on economic model results (no decay, 5% and 10% after Year 5 of model).[4]<br>• Treatment effect size reductions of 0% to 10% in one-unit increments. [13]

	• Intervention effectiveness varied by 10% and 20% higher and lower effects on the incidence rate.[17]<br>• Intervention effects using 20% higher and lower effectiveness values.[5,18]<br>• Effect maintenance scenarios (constant effects that decrease after end of exposure; increasing effects during exposure that decrease after; increasing effects).[5]<br>• Intervention effects decline with simulation time.[2,3,15,21]<br>• More conservative estimates of effectiveness.[20]
Intervention benefits	• Values placed on QALYs/DALYs.[3,5,7,15]
Intervention benefit reach	• Number of people in the sample to receive intervention benefits (e.g. 50% of children to receive benefits)[10,22]<br>• Intervention reach when projecting outcomes reflects intervention uptake in study.[20]<br>• Intervention uptake decreased by 5%, 10% and 25% amongst people in 9th and 10th deciles of deprivation.[4]<br>• % population relapse. [15]<br>• Intervention only benefits certain demographic groups.[5,15]
Discount rate	• Ranged from 0-5% [2,3,7,21]<br>• Ranged from 0-6% [15,23]<br>• Ranged from 0-10% [4]

References

1. Ladapo JA, Bogart LM, Klein DJ, et al. Cost and Cost-Effectiveness of Students for Nutrition and eXercise (SNaX). *Acad Pediatr.* 2016;16(3):247-253.
2. Rush E, Obolonkin V, McLennan S, et al. Lifetime cost effectiveness of a through-school nutrition and physical programme: Project Energize. *Obes Res Clin Pract.* 2014;8(2):e115-e122.
3. Te Velde SJ, Lennert Veerman J, Tak NI, Bosmans JE, Klepp KI, Brug J. Modeling the long term health outcomes and cost-effectiveness of two interventions promoting fruit and vegetable intake among schoolchildren. *Econ Hum Biol.* 2011;9(1):14-22.
4. Mernagh P, Paech A, Coleman K, et al. Assessing the cost-effectiveness of public health interventions to prevent obesity: overview report. Wellington: Health Research Council of New Zealand. 2010.
5. Oosterhoff M, Over EAB, van Giessen A, et al. Lifetime cost-effectiveness and equity impacts of the Healthy Primary School of the Future initiative. *BMC Public Health.* 2020;20(1):1887.
6. Wang LY, Gutin B, Barbeau P, et al. Cost-effectiveness of a school-based obesity prevention program. *J School Health.* 2008;78(12):619-624.
7. Wang LY, Yang Q, Lowry R, Wechsler H. Economic analysis of a school-based obesity prevention program. *Obes Res.* 2003;11(11):1313-1324.
8. Wyatt K, Lloyd J, Creanor S, et al. Cluster randomised controlled trial and economic and process evaluation to determine the effectiveness and cost effectiveness of a novel intervention [Healthy Lifestyles Programme (HeLP)] to prevent obesity in school children. *Public Health Res.* 2018;6(1).
9. Reilly KL, Reeves P, Deeming S, et al. Economic analysis of three interventions of different intensity in improving school implementation of a government healthy

canteen policy in Australia: costs, incremental and relative cost effectiveness. *BMC Public Health.* 2018;18(1):378.

10. Adab P, Barrett T, Bhopal R, et al. The West Midlands ActiVe lifestyle and healthy Eating in School children (WAVES) study: a cluster randomised controlled trial testing the clinical effectiveness and cost-effectiveness of a multifaceted obesity prevention intervention programme targeted at children aged 6-7 years. *Health Technol Assess.* 2018;22(8):1-608.

11. Conesa M, Llaurado E, Aceves-Martins M, et al. Cost-Effectiveness of the EdAl (Educacio en Alimentacio) Program: A Primary School-Based Study to Prevent Childhood Obesity. *J Epidemiol.* 2018;28(12):477-481.

12. Reeves P, Edmunds K, Szewczyk Z, et al. Economic evaluation of a web-based menu planning intervention to improve childcare service adherence with dietary guidelines. *Implement Sci.* 2021;16(1):1.

13. Coffield E, Nihiser A, Carlson S, et al. Shape Up Somerville's return on investment: Multi-group exposure generates net-benefits in a child obesity intervention. *Prev Med Rep.* 2019;16:100954.

14. Brown IHS, Perez A, Li YP, Hoelscher DM, Kelder SH, Rivera R. The cost-effectiveness of a school-based overweight program. *Int J Behav Nutr Phys Act.* 2007;4(47).

15. Graziose MM, Koch PA, Wang YC, Gray HL, Contento IR. Cost-effectiveness of a Nutrition Education Curriculum Intervention in Elementary Schools. *J Nutr Educ Behav.* 2017;49(8):684-691.

16. Haby M, Vos T, Carter R, et al. A new approach to assessing the health benefit from obesity interventions in children and adolescents: The assessing cost-effectiveness in obesity project. *Int J Obes.* 2006;30(10):1463-1475.

17. Kesztyüs D, Lauer R, Kesztyüs T, Kilian R, Steinacker JM. Costs and effects of a state-wide health promotion program in primary schools in Germany - the Baden-Württemberg Study: a cluster-randomized, controlled trial. *PLos ONE.* 2017;12(2):e0172332.
18. Kesztyus D, Schreiber A, Wirt T, et al. Economic evaluation of URMEL-ICE, a school-based overweight prevention programme comprising metabolism, exercise and lifestyle intervention in children. *Eur J Health Econ.* 2013;14(2):185-195.
19. McAuley KA, Taylor RW, Farmer VL, et al. Economic evaluation of a community-based obesity prevention program in children: the APPLE project. *Obesity (Silver Spring).* 2010;18(1):131-136.
20. Kenney EL, Cradock AL, Barrett JL, et al. Cost-Effectiveness of Water Promotion Strategies in Schools for Preventing Childhood Obesity and Increasing Water Intake. *Obesity.* 2019;27(12):2037-2045.
21. Ekwaru JP, Ohinmaa A, Tran BX, Setayeshgar S, Johnson JA, Veugelers PJ. Cost-effectiveness of a school-based health promotion program in Canada: A life-course modeling approach. *PLoS ONE.* 2017;12(5):e0177848.
22. Moodie ML, Herbert JK, de Silva-Sanigorski AM, et al. The cost-effectiveness of a successful community-based obesity prevention program; The Be Active Eat Well Program. *Obesity.* 2013;21(10):2072-2080.
23. An R, Xue H, Wang L, Wang Y. Projecting the impact of a nationwide school plain water access intervention on childhood obesity: a cost-benefit analysis. *Pediatr Obes.* 2018;13(11):715-723.
